# Hydrothermal synthesis and structural optimization of Bi_2_O_3_/Bi_2_WO_6_ nanocomposites for synergistic photodegradation of Indigo Carmine dye

**DOI:** 10.1038/s41598-025-01925-z

**Published:** 2025-05-18

**Authors:** Mostafa A. Sayed, S. M. A. El-Gamal, M. Ramadan, Fatma M. Helmy, Alaa Mohsen

**Affiliations:** 1 Chemistry Department, Faculty of Science, Ain Shams University, Cairo 11566, Egypt; 2 Faculty of Engineering, Ain Shams University, Cairo, Egypt

**Keywords:** Bi_2_O_3_/Bi_2_WO_6_ nanocomposites, Hydrothermal synthesis, Structural optimization, photodegradation, Indigo dye, Chemistry, Materials science, Nanoscience and technology

## Abstract

Future research directions aim to optimize the efficiency and sustainability of bismuth-based semiconductors for environmental remediation. In this study, potent Bi_2_O_3_/Bi_2_WO_6_ composites were synthesized via a facile in situ hydrothermal-assisted impregnation of Bi^3+^ onto WO_3_ nano-substrate. Comprehensive characterization using HR-TEM, SEM-EDX, PXRD, XPS, FTIR, PL, and DRS confirmed the structural, morphological, and optical properties of the synthesized materials. The optimized Bi_2_O_3_/Bi_2_WO_6_ heterojunction exhibited significantly enhanced photocatalytic activity under visible-light (λ > 350 nm) compared to pristine Bi_2_O_3_ and WO_3_, effectively degrading Indigo Carmine (IC) dye. The UV–Vis spectroscopy and chemical oxygen demand (COD) analyses validated the degradation efficiency. A detailed photocatalytic mechanism was proposed based on trapping experiments, band position calculations, and photoluminescence measurements. Furthermore, the fabricated nanocomposites demonstrated excellent stability and recyclability, highlighting their potential for environmental remediation. This study provides a promising strategy for designing efficient visible-light-driven photocatalysts for wastewater treatment.

**Supplementary Information:**

The online version contains supplementary material available at 10.1038/s41598-025-01925-z.

## Introduction

The increasing discharge of toxic organic pollutants from industrial activities, particularly synthetic dyes, poses severe environmental and health concerns due to their high stability and resistance to conventional wastewater treatment methods^1^. This effluent contains organic and inorganic waste, which can directly affect chemical and biological variables. Notably, the textile sector plays a major role in contributing to these challenges. The textile industry emits many toxic pollutants, including solvents and hazardous textile dyes^2^. The effluent from colored textiles usually has elevated levels of dye compounds. Many of these dyes are harmful, cancerous, and capable of causing genetic mutations. Additionally, the release of dyes can lead to visible environmental changes. Indigo carmine (IC) is a well-established anionic indigoid dye that finds widespread application in various industries, such as textiles, food, and cosmetics^3^. Approximately 20,000 tons of IC dye are manufactured annually to produce blue jeans^4^. It is also utilized as a diagnostic tool for different medical applications. IC dye is hazardous, cancer-causing, and non-biodegradable, posing a significant risk to human health and causing detrimental impacts on the environment^5^. Additionally, it poses a multitude of risks to human health, including respiratory discomfort, gastrointestinal diseases, vision loss, and digestive and cognitive disorders^6^.

Semiconductor photocatalysis’s technology is an intriguing way to effectively eliminate a wide range of organic contaminants, particularly water-polluting dyes, when exposed to UV and/or solar radiation^7,8^. This process allows for completely degrading organic toxins at ambient temperature within a few hours, employing active photocatalysts^9,10^. Photocatalysts are semiconductors that can degrade a wide range of organic compounds, chemicals, and pharmaceutical products by generating reactive radicals and charge carriers upon exposure to light^11^. In recent years, novel photocatalysts have garnered significant attention for their potential in environmental remediation, particularly in addressing challenges such as water purification and air pollution^12^.

The construction of advanced heterojunctions has been widely recognized for its significant impact on the efficient separation and utilization of photo-generated charge carriers in photocatalytic processes^13,14^. In a heterojunction, two semiconductors with different band structures are coupled, allowing for the effective separation of photogenerated electrons and holes. This results in a decrease in charge recombination, which is a major hindrance to photocatalytic efficiency. The band alignment at the interface of the two semiconductors facilitates the transfer of electrons from the conduction band of one material to the conduction band of the other, while holes migrate to the valence band of the second material. This spatial separation of charge carriers enhances their lifetime and increases their participation in photocatalytic reactions, leading to improved overall photocatalytic performance^15^. Numerous studies have demonstrated that heterojunctions not only improve charge carrier dynamics but also enhance the absorption of visible light, further promoting the efficiency of photocatalytic processes^16,17^. Thus, the design of heterojunctions plays a crucial role in optimizing the photocatalytic activity of composite materials, making them highly effective in various environmental and energy-related applications.

Several metal oxide photocatalysts, including CeO_2_, ZrO_2_, ZnO, SnO_2_, TiO_2_, and Bi_2_O_3_, have demonstrated high effectiveness in degrading organic contaminants into benign water and CO_2_ byproducts^18^. Among these, tungsten trioxide (WO_3_) nanoparticles have emerged as a promising candidate due to their stability, non-toxicity, and wide applicability^19^. However, their limited absorption in the visible-light spectrum constrains their photocatalytic performance^20^. Consequently, substantial endeavors have been undertaken to create novel or altered semiconductor photocatalysts that are capable of harnessing visible light. Composite photocatalysts were implemented to optimize the longevity of the electron-hole charge carriers produced by light by minimizing their recombination rate^21^. Additionally, these heterostructure photocatalysts were designed to enhance their activity under visible light. The impregnation of WO_3_ nanoparticles with Bi^3+^ ions has garnered significant attention as a strategy to enhance their photocatalytic activity^22^. The incorporation of Bi^3+^ not only modifies the electronic structure of WO_3_ but also facilitates the formation of bismuth-based semiconductors, which exhibit improved visible-light absorption and charge separation efficiency^23,24^.

Numerous studies have been conducted on bismuth-based semiconductors to improve photocatalysis’s visible-light response, especially for creating various heterojunctions^25,26^. Bismuth tungstate (Bi_2_WO_6_), as an n-type semiconductor, has attracted significant attention in the scientific world due to its unique optical, electrical, and photocatalytic properties, making it a prominent photocatalyst among several bismuth-based counterparts^27^. It belongs to the Aurivillius-phase perovskite oxide category and is composed of fluorite-like [Bi_2_O_2_]^2+^ layers and [WO_4_]^2−^ layers in a 1:1 stoichiometric ratio^28^. It has recently gained significant attention due to its potential as a visible-light-driven photocatalyst with an approximate band gap of 2.8 eV^29^. However, the rapid recombination of light-generated electron-hole pairs reduces energy conversion efficiency, which limits the use of BWO in photocatalysis^30^. The firmly developed heterojunction configuration can be implemented to restrict the recombination of the charge carriers and improve the quantum yield. Consequently, numerous researchers have coupled Bi_2_WO_6_ with various semiconductors, including TiO_2_^31^, Ag_3_PO_4_^32^, BiOBr^25^, V_2_O_5_^33^, BiVO_4_^34^, and ZnO^35,36^.

Bi_2_O_3_ is a p-type semiconductor with a band gap of ~ 2.8 eV. It has been demonstrated to act as a photocatalyst for water splitting and contaminants’ degradation when exposed to visible-light^37^. Nevertheless, the photocatalytic-efficiency of pure Bi_2_O_3_ is also limited due to the elevated chance of recombination between photogenerated holes and electrons^38^. However, the photocatalytic-activity is anticipated to be significantly enhanced by generating a p–n junction structure between the n-type Bi_2_WO_6_ and p-type Bi_2_O_3_^39,40^. After reviewing the available literature, we noticed that there are no published reports on the in situ hydrothermal synthesis of Bi_2_O_3_/Bi_2_WO_6_ heterojunction via the impregnation of WO_3_ nanoparticles with Bi^3+^ ions to degrade IC dye efficiently. The photocatalytic-degradation parameters of the previous semiconductor heterojunctions toward IC dye are presented in Table 1. Thus, this study enables the development of a heterojunction system with synergistic photocatalytic properties by optimizing the interaction between WO_3_ and Bi^3+^ to harness the full potential of the resulting Bi_2_O_3_/Bi_2_WO_6_ composite to achieve efficient photocatalytic degradation of such hazardous industrial dye. The significance of these advances lies not only in their environmental applications but also in their ability to provide scalable, sustainable solutions for large-scale remediation efforts, ultimately advancing the field of photocatalysis for environmental protection.

This work investigated the synthesis of new composites consisting of x% weights of Bi^3+^ and WO_3_ NPs using an in-situ hydrothermal approach. This method has numerous advantages, including outstanding repeatability, narrow size distribution, high yield and elevated product purity. The beneficial features of the fabricated heterojunction were evaluated by examining the degradation of aqueous solutions of Indigo carmine (IC) dye under different light sources. The photocatalytic efficiency of the synthesized composite is compared to the individual components, Bi_2_O_3_ and WO_3_. The mechanism behind the improved photocatalytic efficacy was determined by calculating and analyzing the band structure. In addition, the active species involved in the degradation of IC dye on the optimized composite was examined and the catalyst’s cycling performance was evaluated. Ultimately, a plausible mechanism for the photocatalytic-degradation process was investigated.

**Table 1:** A literature survey on photocatalytic-degradation parameters of semiconductor heterojunctions toward Indigo Carmine (IC) dye.

Material	Synthesis method	Degradation (%)	Irradiation (min.)	Light source	References
Sm^3+^@ ZnS	Reflux	93	210	UV lamp	^41^
CoFe_2_**O**_**4**_/SnO_**2**_	Sol–gel	55	120	Mercury lamp	^42^
Fe^3+^ doped TiO_**2**_	Sol–gel	94	60	Fluorescent bulb	^43^
AgIO_4_**/ZnO**	Chemical precipitation	98	120	sunlight	^44^
WO_3_/CeO_2_	Chemical precipitation	45	120	Halogen lamp	^45^
ZrO_2_/rGO	Chemical precipitation	85	60	Mercury lamp	^46^
Ag@TiO_2_	Sol–gel	97	60	Xenon lamp	^47^
Ag/ZnO	Chemical co-precipitation	95	120	Fluorescent lamps	^48^
Ag_3_**VO**_**4**_/CFB Y@Ag_3_**VO**_**4**_/CFB	Microwave-assisted hydrothermal	95 73	120	UV lamp	^49^
CdS/TiO2	Melt quench technique	88	300	Sunlight	^50^
α-Fe_2_**O**_**3**_/bentonite	Chemical precipitation	18	120	UV lamp Solar light	^51^
Mn & S@TiO_2_	Sol–gel	100	90	Mercury vapor lamp	^52^
Ag/TiO_2_	Sol–gel	97	240	UV lamp	^53^
Se-doped ZnO	Electrochemical	96	480	UV lamp	^54^
ZnO/Nb_2_**O**_**5**_	Electrochemical	97	300	UV lamp	^55^
ZnO-Bi_2_**O**_**3**_-g-C_3_**N**_**4**_/H_2_**O**_**2**_	Combustion	93	180	Xenon lamp	^56^
Bi_2_**S**_**3**_/ZnCo_2_**O**_**4**_	Hydrothermal	90	90	Halogen lamp	^57^

## Experimental section

### Chemicals

The chemicals utilized in the study were employed without any additional purification. The precursors used were Sodium tungstate dihydrate [Na_2_WO_4_·2H_2_O], bismuth nitrate pentahydrate [Bi(NO_3_)_3_.5H_2_O], nitric acid (HNO_3_), acetic acid (CH_3_COOH, HAc), and urea (NH_2_CONH_2_). Polyethylene glycol with a molecular weight of 10,000 (PEG) was employed as a dispersant.

### Synthesis of WO_3_ NPs

To synthesize nano WO_3_, approximately 0.03 mol (9.9 g) of sodium tungstate was dissolved in 150 ml of distilled water. Then, 3.75 g of PEG was added, stirring the mixture for 1 h. Concentrated acetic acid was slowly added drop by drop until the pH reached around 2–3. The resulting white suspension was agitated for a further 2 h at ambient temperature. The solution is transferred into a Teflon-lined cell and placed inside a stainless-steel autoclave in an oven set at 160 °C for 24 h. The product was washed with ethanol and water and then dried at 100 °C for one night. The desiccated sample was ultimately calcinated for 2 h in a muffle furnace at 500 °C, forming a greenish precipitation of WO_3_ NPs. The precipitate was thoroughly pulverized and subsequently utilized for further experimental work.

### Synthesis of Bi_2_O_3_/Bi_2_WO_6_ heterojunctions

The Bi_2_O_3_/Bi_2_WO_6_ photocatalysts were synthesized using the in-situ hydrothermal technique. Initially, a molar ratio of 1.5:1 was established between Bi_2_O_3_ and WO_3_. Subsequently, 14.55 g of Bi(NO_3_)_3_.5H_2_O were dissolved in 100 ml of acetic acid (HAc). This solution was then slowly added drop by drop to 50 mL of deionized water containing 2.32 g of highly dispersed WO_3_ NPs. The resulting suspension was mixed with urea in a 1:5 molar ratio (50 ml) under vigorous magnetic stirring at room temperature. The mixture was agitated using a magnetic stirrer for 30 min at an ambient temperature. The suspension was then transferred to 250 ml Teflon-lined stainless-steel autoclaves with a capacity of ~ 75% of their maximum volume. The autoclave was then heated to a specific temperature of 160 °C for 24 h. After the autoclaves cooled to room temperature, the precipitate was separated using centrifugation at 3000 rpm for 5 min. The sample was subjected to three washing cycles with deionized water and ethanol to eliminate any potentially interfering ionic species. The resulting mixture was subjected to drying at a temperature of 100 °C for 24 h. The dried sample was subjected to calcination for 2 h in a muffle at 500 °C, forming bright yellow precipitates to harvest the Bi_2_O_3_/Bi_2_WO_6_ composite, labelled as a BW1 sample. The inclusion of the (Bi_2_WO_6_) phase in the composite is attributed to the effectiveness of hydrothermal treatment (HT) in the presence of urea (pH ≈ 10), as indicated by the chemical reaction below^58^:

$$2{\text{Bi}}({\text{NO}}_{3} )_{3} \cdot 5{\text{H}}_{2} {\text{O}}_{{({\text{aq}}.)}} + {\text{WO}}_{{3({\text{s}})}} \xrightarrow[{{\text{pH}} \sim 10}]{{{\text{HT}}}}{\text{Bi}}_{2} {\text{WO}}_{{6({\text{s}})}}$$

**Fig. 1:**
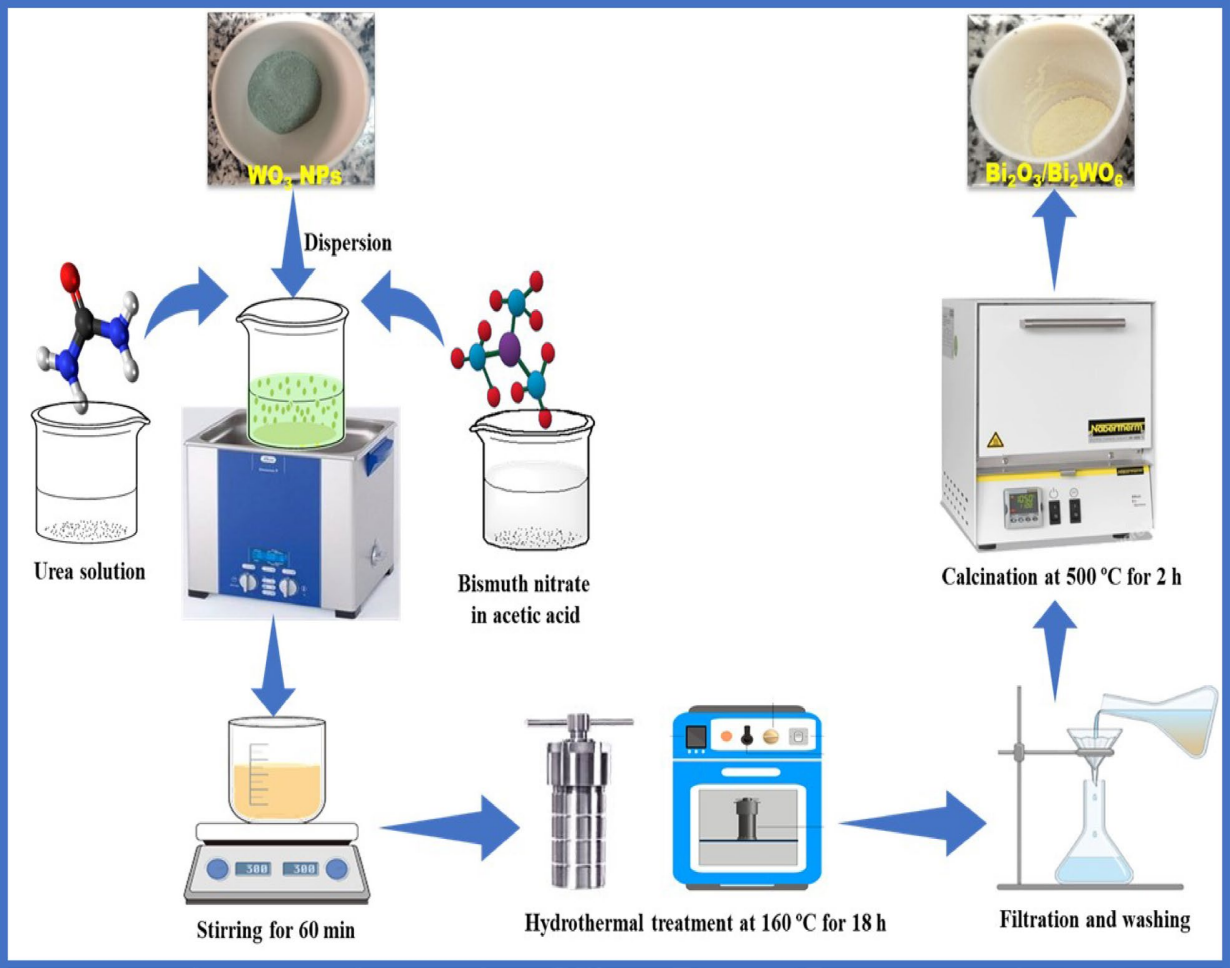
Illustrative scheme for the preparation of Bi_2_O_3_/Bi_2_WO_6_ heterostructure.

For comparison, composites with molar ratios of Bi_2_O_3_:WO_3_ at 1:1 and 0.5:1 were synthesized and labeled as BW2 and BW3 samples, respectively. Besides, Bi_2_O_3_ powders were synthesized via a hydrothermal technique using identical preparation conditions indicated earlier, excluding adding WO_3_ NPs. The synthesis-steps of Bi_2_O_3_/Bi_2_WO_6_ heterostructures were summarized in Fig. 1.

### Photocatalytic performance experiments

The photocatalytic performance of the fabricated nanomaterials was evaluated in a photoreactor equipped with UVA (6w, 365 nm, ~ 1100 mW/cm^2^), UVC (6w, 254 nm, ~ 1050 mW/cm^2^), and LED (15w, λ ≥ 380 nm, ~ 1350 mW/cm^2^) light sources at room temperature (~ 30 °C). The degradation of the Indigo carmine (IC) dye (C_16_H_8_N_2_Na_2_O_8_S_2_, 466.36 g/mol) was monitored during the experiment. For every degradation experiment, a 100 ml aqueous solution containing IC was carefully poured into the beaker, maintaining a consistent initial concentration of 50 mg/l (50 ppm). Then, a specific amount of the photocatalyst (50 mg) was added to the solution while continuously stirring it with a magnetic stirrer for 30 min. This step was carried out in a dark environment to ensure that the adsorption-desorption equilibrium was achieved before proceeding with the photocatalytic-degradation. The light was then activated to initiate the degradation process. At regular time intervals of 10 min, a 2 mL portion of the suspension was carefully extracted and subjected to centrifugation at 5000 rpm for 3 min. The solution was then analyzed using a UV–Vis spectrophotometer to determine its absorbance maximal at 610 nm. A calibration curve was employed to ascertain the IC concentration based on the peak absorbance. An extensive examination of multiple factors, such as pH, catalyst dosage, contact duration, initial dye concentration, and trapping agents, was successfully conducted. The degradation efficiency of the synthesized catalysts has been evaluated using Eq. (1):

1 $$\:\mathbf{D}\mathbf{e}\mathbf{g}\mathbf{r}\mathbf{a}\mathbf{d}\mathbf{a}\mathbf{t}\mathbf{i}\mathbf{o}\mathbf{n}\:\mathbf{e}\mathbf{f}\mathbf{f}\mathbf{i}\mathbf{c}\mathbf{i}\mathbf{e}\mathbf{n}\mathbf{c}\mathbf{y}\:(\mathbf{D},\mathbf{\%})=\frac{{\mathbf{A}}_{\mathbf{o}}-{\mathbf{A}}_{\mathbf{t}}}{{\mathbf{A}}_{\mathbf{o}}}\times\:100$$

where, A_○_ and A_t_ are the absorbance readings of IC before and after exposure to light, respectively.

According to the Hinshelwood–Langmuir approach, the photodegradation of organic toxins usually follows the pseudo-first-order kinetics, so the photo-decolorization rate of IC dye was studied by Eq. (2):

2 $$\:\mathbf{l}\mathbf{n}\frac{{\mathbf{C}}_{\mathbf{o}}}{{\mathbf{C}}_{\mathbf{t}}}=\mathbf{k}\mathbf{t}$$

where (C_0_) is the initial concentration and (C_t_) is the concentration at any time, t. The rate constants (k) were estimated from the slopes of the straight-line segment of the plots of ln(C_0_/C_t_) versus t for each reaction.

The COD of the dye solution was measured before and after the photolysis reactions at regular irradiation intervals to determine if only discoloration or complete dye mineralization takes place during the photodegradation process using the dichromate-oxidation method^59^. The mineralization proficiency was determined by Eq. (3):

3 $$\:\mathbf{M}\mathbf{i}\mathbf{n}\mathbf{e}\mathbf{r}\mathbf{a}\mathbf{l}\mathbf{i}\mathbf{z}\mathbf{a}\mathbf{t}\mathbf{i}\mathbf{o}\mathbf{n}\:\mathbf{e}\mathbf{f}\mathbf{f}\mathbf{i}\mathbf{c}\mathbf{i}\mathbf{e}\mathbf{n}\mathbf{c}\mathbf{y}\:\left(\mathbf{M},\mathbf{\%}\right)=\frac{{\mathbf{C}\mathbf{O}\mathbf{D}}_{\mathbf{i}}-{\mathbf{C}\mathbf{O}\mathbf{D}}_{\mathbf{f}}}{{\mathbf{C}\mathbf{O}\mathbf{D}}_{\mathbf{i}}}\:\times\:100$$

COD_i_ was the value measured before exposure to light, while COD_f_ was the COD-value measured after exposure to light at regular time intervals. The measurements were done three times, and the mean values were noted.

### Detection of active species

Quenching tests were conducted to provide insight into the active species and potential photocatalytic pathways. The experiment followed a similar procedure to the photocatalytic test, including adding scavengers to the reaction solutions before introducing the photocatalyst. The photodegradation systems utilized 0.5 mmol of p-benzoquinone (p-BQ), ammonium oxalate (AO), potassium dichromate (K_2_Cr_2_O_7_), and isopropyl alcohol (IPA) as scavengers. These scavengers were employed to effectively suppress the activity of superoxide radicals (·O^2−^), photo-holes (h^+^), photoelectrons (e^−^), and hydroxyl radicals (·OH), respectively.

## Results and discussion

### Morphological and structural features of synthesized materials

To identify the role of synthesized nanomaterials (WO_3_, Bi_2_O_3_ and BW1) and measure their efficiencies in photocatalytic degradation for Indigo carmine (IC) dye, their phase composition, degree of crystallinity, functional group, morphology, purity, and crystal size were examined using XRD, FTIR, SEM/EDX and HRTEM/SAED. The composite BW1 was specifically selected for a comprehensive characterization while disregarding the other composites (BW2 and BW3) because the BW1 composite exhibited the highest catalytic activity in degrading the target dye (IC), which will be explained in more detail later.

#### Phase identification

**Fig. 2:**
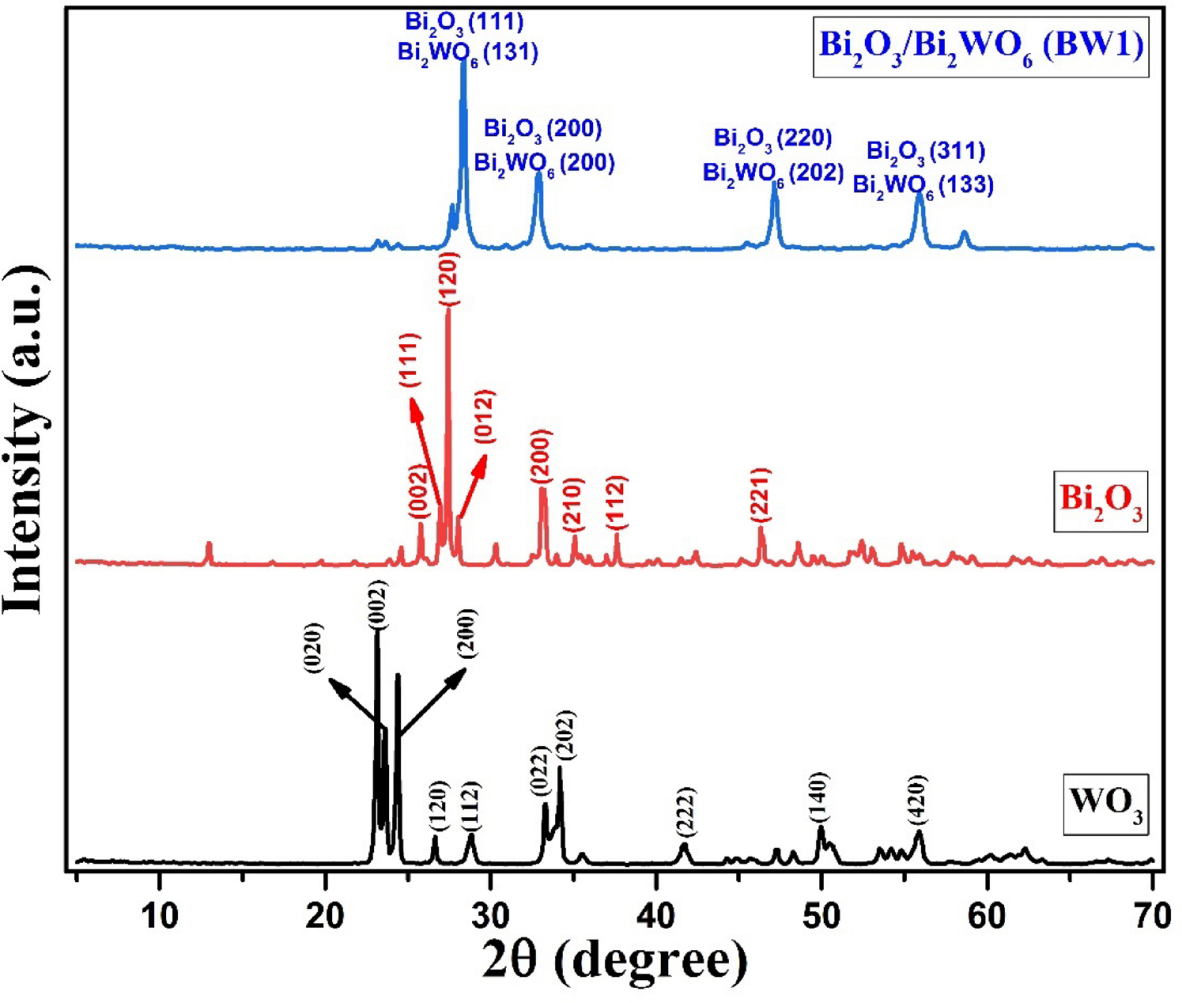
XRD-patterns of synthesized nanomaterials.

Powder X-ray diffraction was utilized to ascertain the phase composition of the solid powders and to confirm the successful synthesis of the synthesized materials^60,61^. The XRD analysis (Fig. 2) shows that all synthesized nanomaterials have high purity. The XRD-diffractogram of WO_3_ NPs displays several distinguish peaks having different crystallographic-planes with specific reflection-peaks (hkl) at 2Ɵ= 23.11° (002), 23.57° (, 24.35° (200), 26.59° (120), 28.96° (121), 33.25° (022), 34.15° (202), 41.64° (221), 49.89° (140) and 55.89° (420), matching with Hatel and Baitoul^62^. These peaks refer to the formation of pure monoclinic WO_3_ NPs, according to PDF# 01-072-0677. The peaks observed in XRD-diffractogram of Bi_2_O_3_ NPs at 2Ɵ= 25.75° (002), 26.91° (111), 27.39° (120), 28.01° (012), 33.26° (200), 35.04° (210), 37.62° (112) and 46.34° (221) are related to monoclinic α-Bi_2_O_3_ NPs according to PDF# 01-071-2274, as identified by Oudghiri-Hassani et al.^63^. For the Bi2O3/Bi2WO6 heterostructure, four prominent peaks are detected at 2Ɵ= 28.37, 32.85, 47.11, and 55.93°. At each peak, there are two overlapped phases are identified, which are cubic Bi_2_O_3_ (PDF# 01-076-2478 with reflection-peaks 111, 200, 220 and 311, respectively) and orthorhombic Bi_2_WO_6_ (PDF# 01-073-1126 with reflection-peaks 131, 200, 202 and 133, respectively), in line with Singh et al.^64^. It is important to notice that the overlap of the diffraction peaks of Bi_2_O_3_ and Bi_2_WO_6_ in the BW1 composite indicates the effective development of Bi_2_O_3_/Bi_2_WO_6_ heterostructure via this synthesis route^65^. Moreover, the absence of any additional phases is confirmed, indicating the samples’ great purity.

From XRD-analysis, the degree of crystallinity was measured using Match! 3 version 3.15 software. All the samples exhibit a satisfactory level of crystallinity. It was found that the degree of crystallinity of WO_3_ NPs > Bi_2_O_3_ NPs > Bi_2_O_3_/Bi_2_WO_6_ heterostructure; their values are 81.22%, 77.12%, and 76.95%, respectively. Also, the average crystal-size was calculated using Debye–Scherrer’s equation (D = Kλ/βcosƟ), where D: crystal-size, K: shape-factor (equal 0.92), λ: wavelength of X-ray (1.5406 A°), β: full-width at half-maximum, FWHM), and θ: scattering-angle. It was found that the crystal sizes of WO_3_ NPs ranged from 9.58 to 42.88 nm with an average size of 25.67 nm, Bi_2_O_3_ NPs ranged from 7.78 to 50.71 nm with an average size of 36.71 nm, and Bi_2_O_3_/Bi_2_WO_6_ composite ranged from 6.88 to 31.22 nm with an average size of 18.10 nm. The smaller particle size of the Bi_2_O_3_/Bi_2_WO_6_ composite with respect to WO_3_ and Bi_2_O_3_ may be attributed to the acting of the Bi_2_WO_6_ nanoparticles as a nucleation site for Bi_2_O_3_^66^. This results in increasing dispersion and thus reducing the particle size^67^. Also, these results match the degree of crystallinity; the low degree of crystallinity refers to the low particle size. Kominami et al.^68^ and Sudrajat and Sujaridworakun^69^ reported that the degree of crystallinity of small particles is lower than that of large particles. Moreover, the high surface area of Bi_2_O_3_/Bi_2_WO_6_ composite with respect to WO_3_ and Bi_2_O_3,_ as will be illustrated below (“Textural parameters studies”), is strong evidence of the smaller particle size of the nanocomposite.

#### Functional group identification

**Fig. 3:**
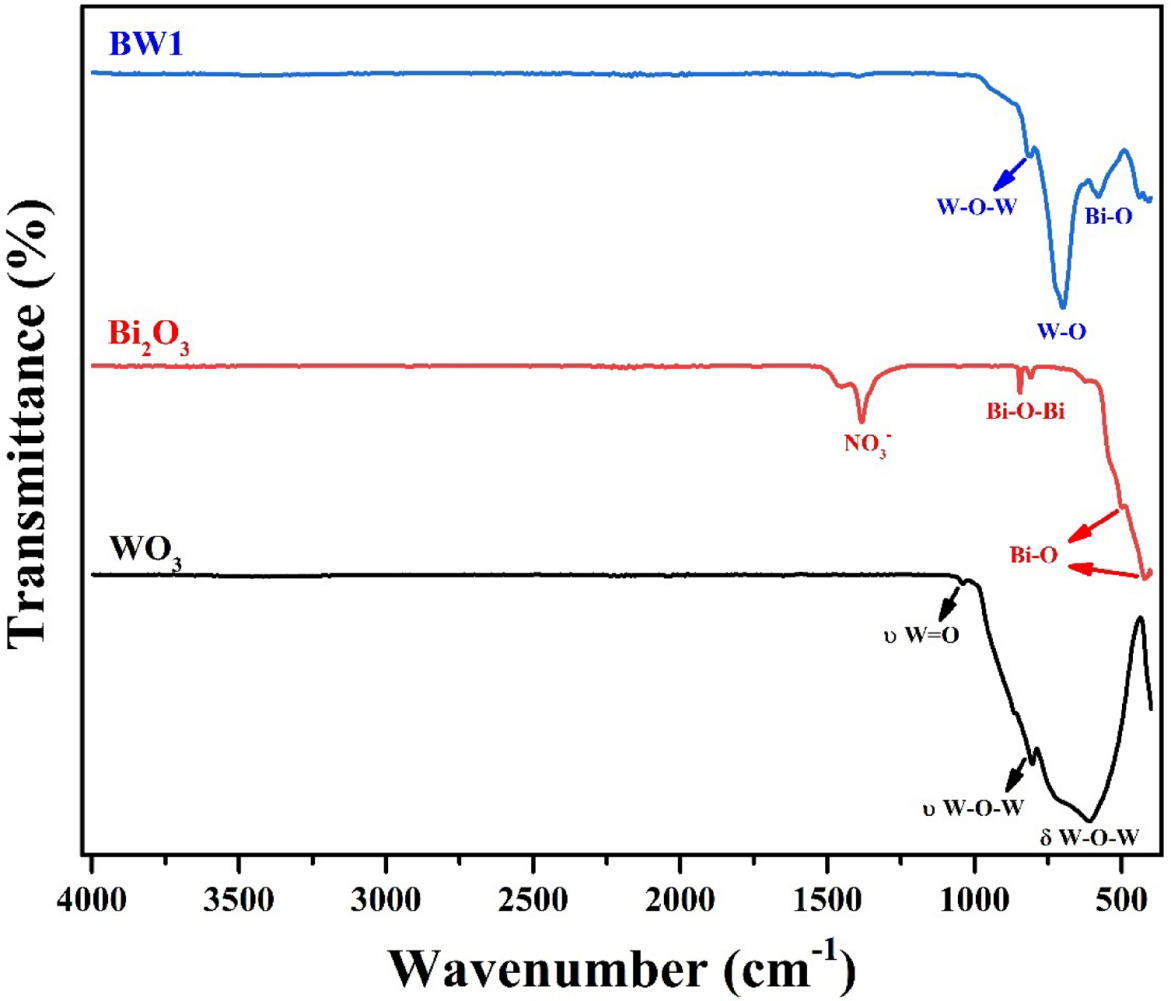
FTIR-spectra of synthesized nanomaterials.

The FTIR-spectra of WO_3_, Bi_2_O_3_ and Bi_2_O_3_/Bi_2_WO_6_ are represented in Fig. 3 to confirm their structure by identifying various bonds’ existence. The spectrum of each sample displays several transmittance-bands at different wavenumbers. The WO_3_ NPs’ spectrum demonstrates the presence of three bands affiliated to δ W-O-W stretching, υ W-O_inter_-W bridge stretching and υ W =O symmetrical stretching, which are centered at 624, 803 and 1042 cm^− 1^, aligning well with results from earlier comparable studies^19,70^. For Bi_2_O_3_ NPs, three bands are observed associated with vibrations of the Bi–O bond at 430 and 496 cm^− 1^ and Bi–O–Bi stretching at 843 cm^− 1^, aligning with Li et al.^71^. The band detected at 1374 cm^− 1^ may be correlated to N–O bending vibrations of NO_3_^−^ resulting from the Bi_2_(NO_3_)_3_.5H_2_O precursor used in the preparation procedure, as described by Priscilla et al.^72^. Regarding Bi_2_O_3_/Bi_2_WO_6_ heterostructure, absorption bands in the range 500–1000 cm^− 1^ are assigned to the metal-oxygen stretching bond. The Bi-O stretching, W-O stretching and W–O–W bridge stretching bands are found at 585, 705 and 815 cm^− 1^, respectively, as identified by Salari^73^, Zhou et al.^74^, Song et al.^75^ and Dong et al.^76^. Furthermore, the bands observed within this region correspond to the orthorhombic phase of Bi_2_WO_6_^77^. These results affirmed the formation of pure WO_3_ NPs, Bi_2_O_3_ NPs and Bi_2_O_3_/Bi_2_WO_6_ heterostructure.

#### Surface morphological analysis

**Fig. 4:**
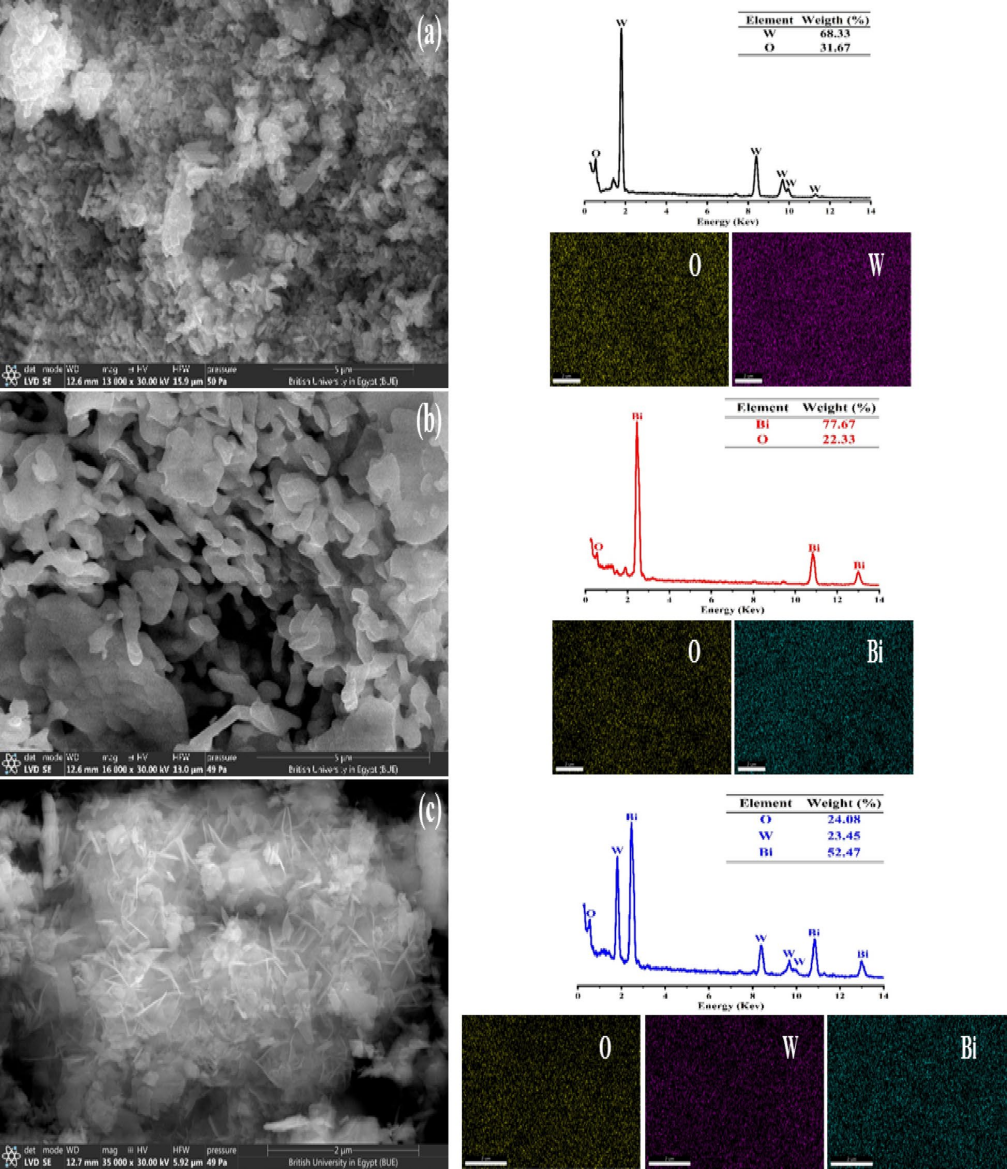
SEM/EDX/EDS-mapping of synthesized nanomaterials: (**a**) WO_3_ (**b**) Bi_2_O_3_, and (**c**) BW1.

**Fig. 5:**
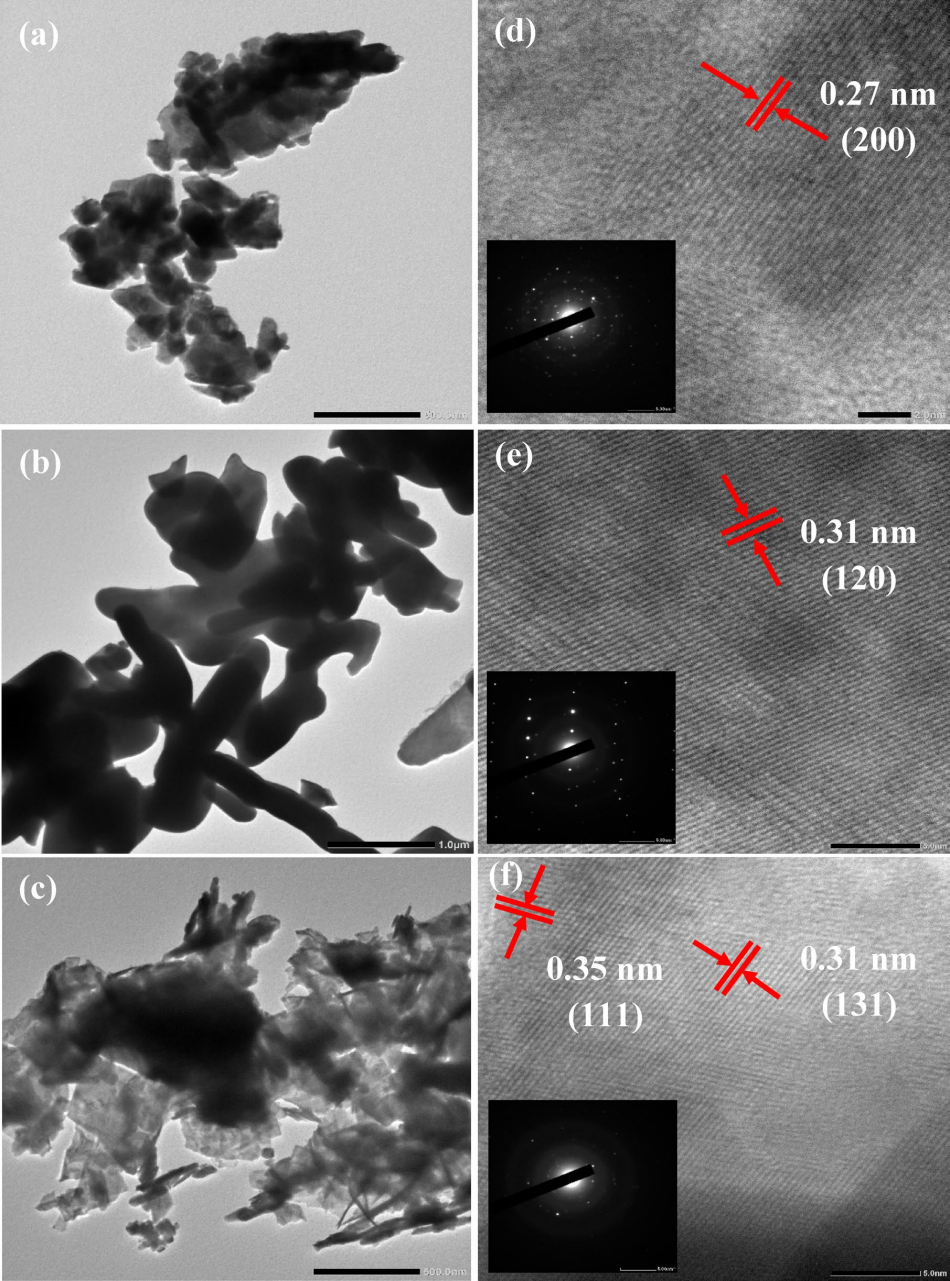
TEM images (**a**–**c**) and their corresponding HR-TEM/SAED patterns (**d**–**f**) of synthesized nanomaterials; (**a**) WO_3_, (**b**) Bi_2_O_3_, and (**c**) BW1.

The morphology, purity, and particle size distribution of synthesized materials were investigated using SEM/EDS-mapping/EDX (Fig. 4) and HR-TEM/SAED (Fig. 5). The SEM-micrographs reveal that the WO_3_ NPs, Bi_2_O_3_ NPs and Bi_2_O_3_/Bi_2_WO_6_ heterostructure have spheroid-like structure (matched with Sayed et al.^19^), worm-like structure (matched with Moghadam and Farzaneh^78^) and nano-flakes developed hierarchical flower-like structure (matched with Dong et al.^76^), respectively. The spheroid particle characteristics of WO_3_ disappeared and were transformed into a newly generated hierarchical structure in the composite. Furthermore, the presence of substantial clusters of Bi_2_WO_6_ and the accumulation of nanosized B_i2_O_3_ particles on the outer surface provide unambiguous evidence of the creation of a Bi_2_O_3_/Bi_2_WO_6_ composite (Fig. 4c). The elemental analysis using EDS-mapping/EDX shows a uniform distribution of the elements as well as the presence of W and O only in WO_3_ NPs, Bi and O only in Bi_2_O_3_ NPs and W, Bi and O in Bi_2_O_3_/Bi_2_WO_6_ heterostructure. The absence of impurities in the samples confirms the high purity level of synthesized samples. The TEM images (Fig. 5a,c) revealed the presence of highly agglomerated nanosized particles, which corresponded to the particle morphologies observed in the SEM images (Fig. 4). The significant agglomeration can be ascribed to the highly minute particle size dispersion in the examined samples^79^. On the other hand, HR-TEM/SAED images/patterns (Fig. 5d–f) provide information on atoms on the NPs’ crystal lattice by measuring the interplanar spacing (d-spacing, nm) and identifying the lattice fringes. The d-spacing of WO_3_ NPs is 0.27 nm, corresponding to (200) crystallographic-plane of monoclinic WO_3_ NPs (PDF# 01-072-0677). The d-spacing of Bi_2_O_3_ NPs is 0.31 nm, relating to (120) crystallographic-plane of monoclinic α-Bi_2_O_3_ NPs (PDF# 01-071-2274). In BW1 composite (Fig. 5f), there are two distinct patterns of lattice fringes with interplanar d-spacings of 0.31 nm and 0.35 nm, correlating to (131) crystallographic-plane of orthorhombic Bi_2_WO_6_ NPs (PDF# 01-073-1126) and (111) crystallographic-plane of cubic Bi_2_O_3_ NPs (PDF# 01-076-2478), respectively, confirming the successful formation of heterostructure composite. The estimated d-spacing values and their related lattice planes from this pattern are accurately fitted with the XRD data. Furthermore, the SAED patterns revealed that the spot analysis areas of all the manufactured materials exhibited a distinct lack of ambiguity, indicating a significant level of crystallinity, particularly in polycrystalline forms. These findings align with those obtained from the PXRD study depicted in Fig. 2.

#### XPS analysis

**Fig. 6:**
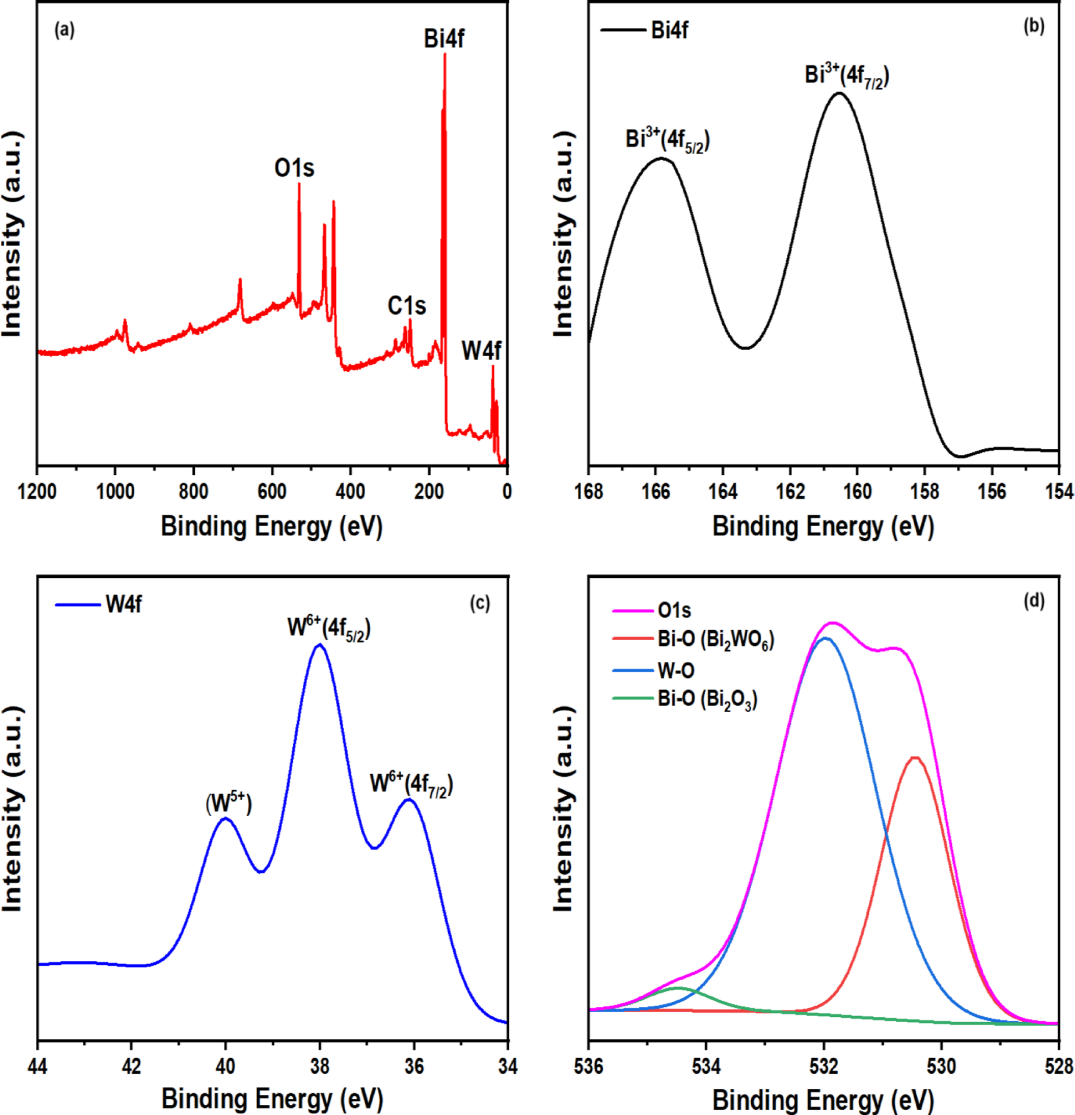
XPS-spectra of BW1 composite: (**a**) survey, (**b**) Bi4f, (**c**) W4f, and (**d**) O1s.

XPS-analysis was performed quantitatively on the fabricated BW1 heterostructure. The full survey and high-resolution spectra for the Bi4f, W4f, and O1s species (Fig. 6) indicated the presence of only O, Bi, and W elements in the sample. The XPS-spectrum of the W4f zone displayed peaks at binding energies of 36.1 eV (W 4f_7/2_) and 38.0 eV (W 4f_5/2_), characteristic of the W^6+^ oxidation state^80,81^. However, low-intensity peaks at 40.0 eV may have resulted from the existence of W^5+^ and oxygen vacancies in the Bi_2_WO_6_ matrix^82^. The deconvoluted Bi4f spectrum exhibited peaks at binding energies of 165.8 eV (Bi 4f_5/2_) and 160.5 eV (Bi 4f_7/2_), confirming the presence of the Bi^3+^ state^83–85^. Although these binding energies are indicative of Bi^3+^ and W^6+^ species, they do not precisely match those observed in pure Bi_2_O_3_ or Bi_2_WO_6_, suggesting the formation of a unique chemical environment in the BW1 heterostructure. This observation also implies forming an interfacial structure, resulting in a modification of the constituent elements’ local environments and electron densities^38^. The O 1s XPS-spectrum could be deconvoluted into three peaks centered at 530.5, 532, and 534.5 eV, which were attributed to Bi–O and W–O bonds in Bi_2_WO_6_ and Bi–O bonds in Bi_2_O_3_, respectively^86–89^. Besides, oxygen vacancies within the composite lattice are observable through an asymmetric profile of the O1s profile^90^. In contrast, the XPS-analysis of sample BW1 revealed that the surface contains 23.9 atom% Bi^3+^ and 9.6 atom% W^6+^. The proportion of bismuth to tungsten atoms is approximately 2.5, exceeding the stoichiometric ratio found in Bi_2_WO_6_. The finding shows that both Bi_2_O_3_ and Bi_2_WO_6_ species are present in the BW1 composite, which agrees with the findings obtained from XRD, HR-TEM, and SEM analyses.

#### Textural parameters studies

**Fig. 7:**
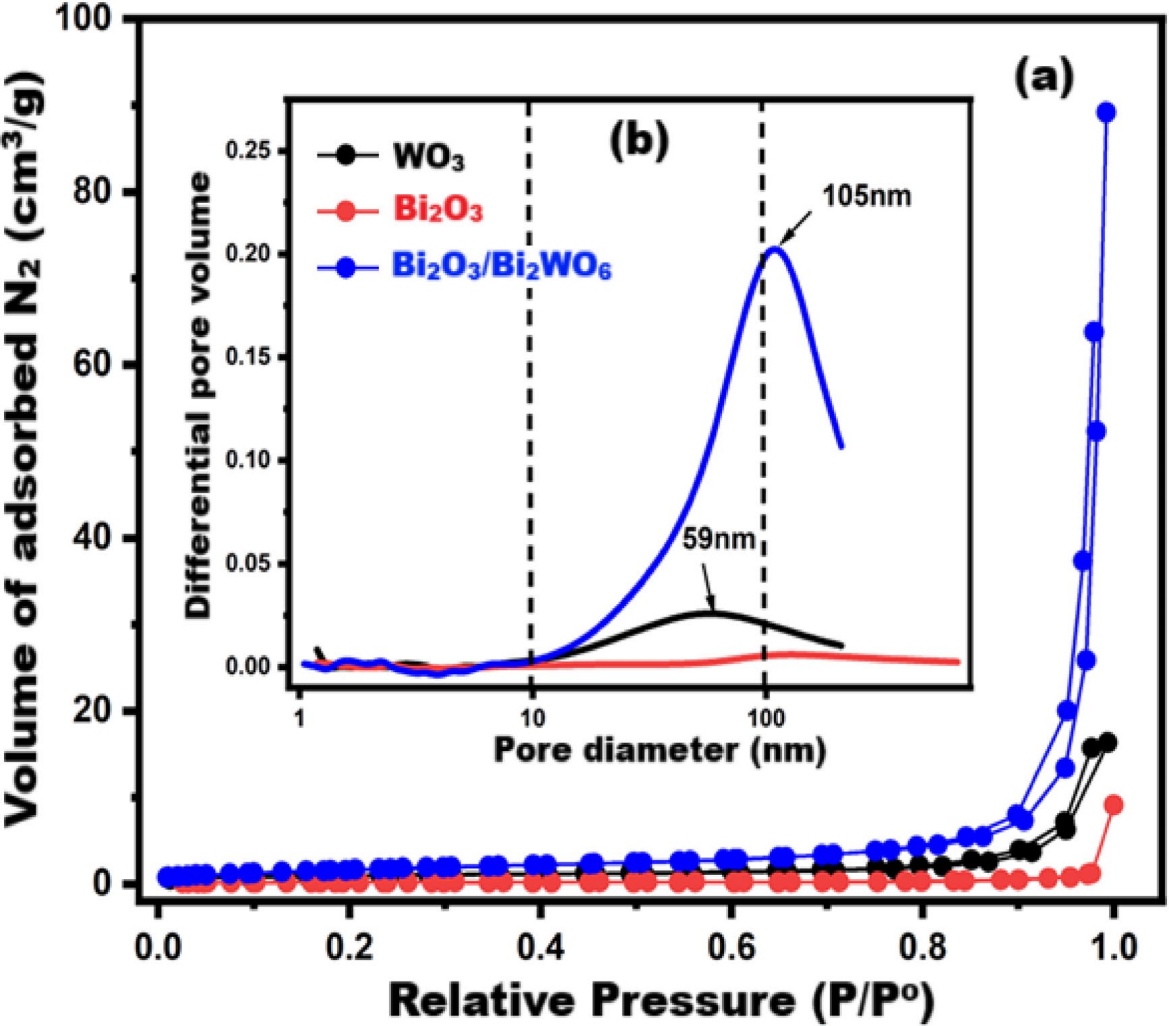
(**a**) N_2_-adsoption/desorption isotherms (**b**) Pore size distributions of synthesized WO_3_, Bi_2_O_3_ and Bi_2_O_3_/Bi_2_WO_6_.

The texture parameters were analyzed via Brunauer–Emmett–Teller model (BET) and Barrett–Joyner–Halenda model (BJH). Figure 7a,b displays N_2_-adsoprtion/desorption isotherms and BJH-pore size distribution curves for the prepared metal oxides. According to IUPAC classification, Fig. 7a confirms that WO_3_ and Bi_2_O_3_ obey Type III isotherm while Bi_2_O_3_/Bi_2_WO_6_ obey Type III/H3 hysteresis-loop reflecting the meso-porous nature of the prepared composite. The hysteresis is attributed to the capillary condensation phenomena^91,92^ inside the mesoporous system of Bi_2_O_3_/Bi_2_WO_6_ of 105 nm maximum and pore diameter, as clarified in Fig. 7b. Moreover, Table 2 affirms that Bi_2_O_3_/Bi_2_WO_6_ NPs possess the highest textural parameters as compared with single oxides as the BET-specific surface area (S.S.A), monolayer capacity (Vm), and total pore volume (Vt) are 6.57 m^2^/g, 1.51 cm^3^/g, and 0.127 cm^3^/g, respectively. Thus, these outcomes indicate that the prepared Bi_2_O_3_/Bi_2_WO_6_ NPs will have a distinct photo-catalytic performance compared to WO_3_ and Bi_2_O_3_ NPs and will hence act as effective active centers for the degradation of the target dye^93^.

**Table 2:** Textural parameters of the synthesized materials.

Material	S.S.A (m^2^/g)	Vm (cm^3^/g)	Vt (cm^3^/g)	dpmax (nm)	Av pore diameter (nm)	Isotherm type
WO_3_	3.08	0.708	0.024	59	30	III
Bi_2_O_3_	0.66	0.152	0.009	105	100	III
Bi_2_O_3_/Bi_2_WO_6_	6.57	1.51	0.127	105	69	III/H3

### Optical properties

#### UV–Vis diffuse reflectance spectral studies (UV-DRS)

**Fig. 8:**
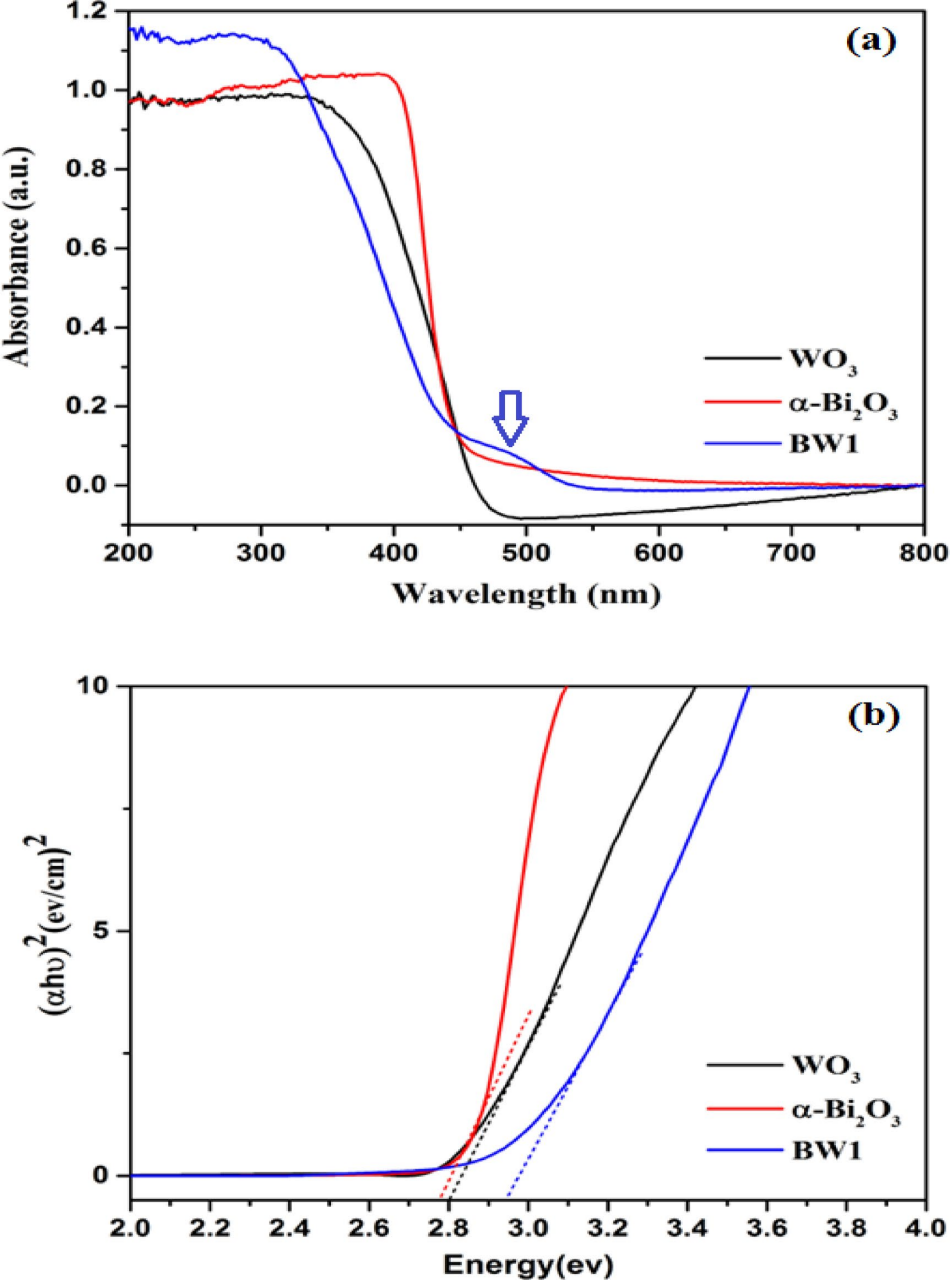
Solid-state UV–Vis absorption spectra (**a**) and Tauc’s Plot (**b**) of the fabricated nanomaterials.

For evaluating the photocatalytic-performance of the synthesized photocatalysts, it is crucial to consider the specific range of wavelengths of light that they can absorb^94^. Hence, the optical characteristics of the synthesized nanocomposite (BW1) were investigated using UV-DRS analysis and compared to the pristine WO_3_ and α-Bi_2_O_3_ nanoparticles, as depicted in Fig. 8. The DRS spectra of pure WO_3_, α-Bi_2_O_3_, and BW1 composite exhibited an optical absorption edge at wavelengths of 452 nm, 456 nm, and 439 nm, respectively, as displayed in Fig. 8a. This demonstrates their abilities to absorb visible light. Nevertheless, the BW1 composite exhibits absorption shoulders at approximately 490 nm, suggesting that this catalyst has a greater capacity for absorbing light across a wide range of visible regions. The enhanced visible-light absorption of BW1 composite material can be attributed to the surface plasmon band heterojunction by the well-distributed Bi_2_O_3_ nanoparticles^95^. Besides, upon the amalgamation of the two semiconductors, Bi_2_O_3_/Bi_2_WO_6_ heterojunction (BW1) exhibits heightened absorption within the visible-light spectrum, surpassing that of pure α-Bi_2_O_3_ or WO_3_ components. The direct bandgap energy (Eg) of the synthesized semiconductors was determined by fitting the absorption data to the direct transition equation (Tauc’s equation)^96^:

4 $$\:{\left(\varvec{\upalpha\:}\mathbf{h}\varvec{\upupsilon\:}\right)}^{2}=\mathbf{A}\left(\mathbf{h}\varvec{\upupsilon\:}-{\mathbf{E}}_{\mathbf{g}}\right)$$

where α is the absorption coefficient, hυ is the photon energy, Eg is the direct bandgap of the material and A is constant. The bandgap energies of synthesized materials have been measured by plotting (αhυ)^2^ as a function of photon energy (hυ) and extrapolating the linear portion of the curve to absorption equal to zero^97^. The plots of (αhυ)^2^ versus (hυ) are shown in Fig. 8b and the corresponding bandgap energies depending upon these plots were determined to be 2.8 eV, 2.78 eV, and 2.93 eV for WO_3_, α-Bi_2_O_3_, and BW1 composite, respectively. The increase in the bandgaps upon the formation of the Bi_2_O_3_/Bi_2_WO_6_ nanocomposites, as observed in our study, can be attributed to the interactions between the Bi_2_O_3_ and Bi_2_WO_6_ components. Specifically, the formation of the heterojunction can lead to a shift in the electronic structure of both semiconductors^40,98^. The interface between the two materials can result in band bending, which may affect the electronic states and the absorption of light. Furthermore, the increase in bandgap could also be a result of the quantum confinement effects in the nanocomposite, as the size of the particles decreases during synthesis^99^. Clearly, the integration of Bi_2_O_3_ nanoparticles into Bi_2_WO_6_ improves its ability to absorb visible light. This can be attributed to the mutual photosensitization of Bi_2_O_3_ and Bi_2_WO_6_ surfaces^100^.

#### Photoluminescence (PL) measurements

**Fig. 9:**
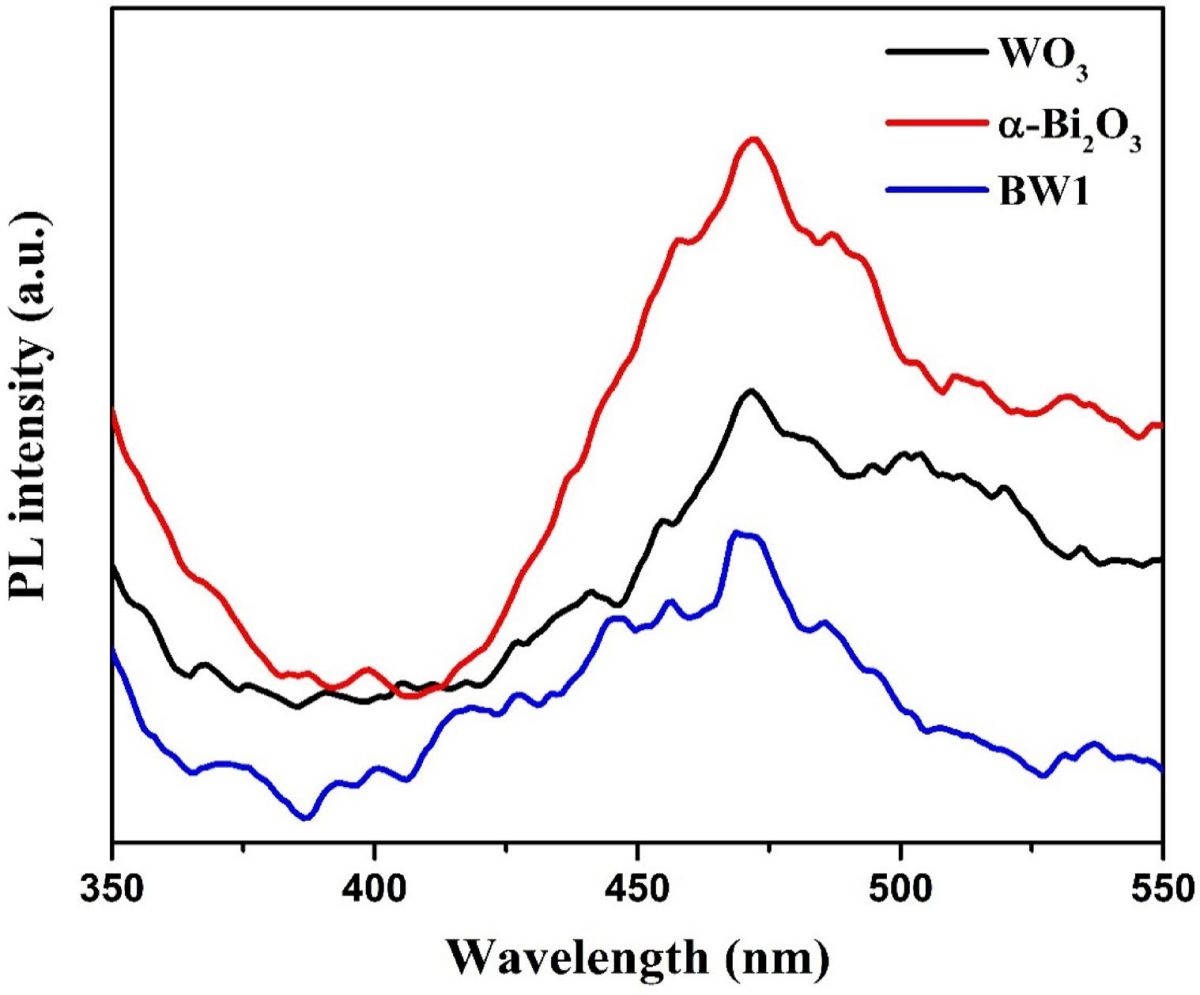
Photoluminescence spectra (λ_ex_ = 300 nm) of the synthesized nanomaterials.

Photoluminescence (PL) experiments were conducted on all the manufactured solid samples to examine the photogenerated electron-hole pairs’ lifespan. The PL spectra are directly associated with the transfer dynamics of the photoexcited electrons and generated holes. The samples exhibited green emission bands spanning a wavelength range of 450 to 550 nm (Fig. 9). The recombination of electron-hole pairs is responsible for the intense emission bands observed in bare WO_3_ and Bi_2_O_3_ nanoparticles^101,102^. In contrast, the PL emission intensity of the composite Bi_2_O_3_/Bi_2_WO_6_ (BW1) is considerably lower compared to individual precursors, suggesting a reduction in the photogenerated charges’ recombination^103^. This reduction causes a significant increase in the lifetime of the reactive radicals that are produced^104^. Thus, the electron and hole pairs generated by light absorption are less likely to be recombined because the charge carriers are spatially separated across the heterojunction, which leads to a decrease in PL intensity despite the relative increase in bandgap^15,105^.

### Photocatalytic performance of synthesized nanomaterials

#### Effect of catalyst type

**Fig. 10:**
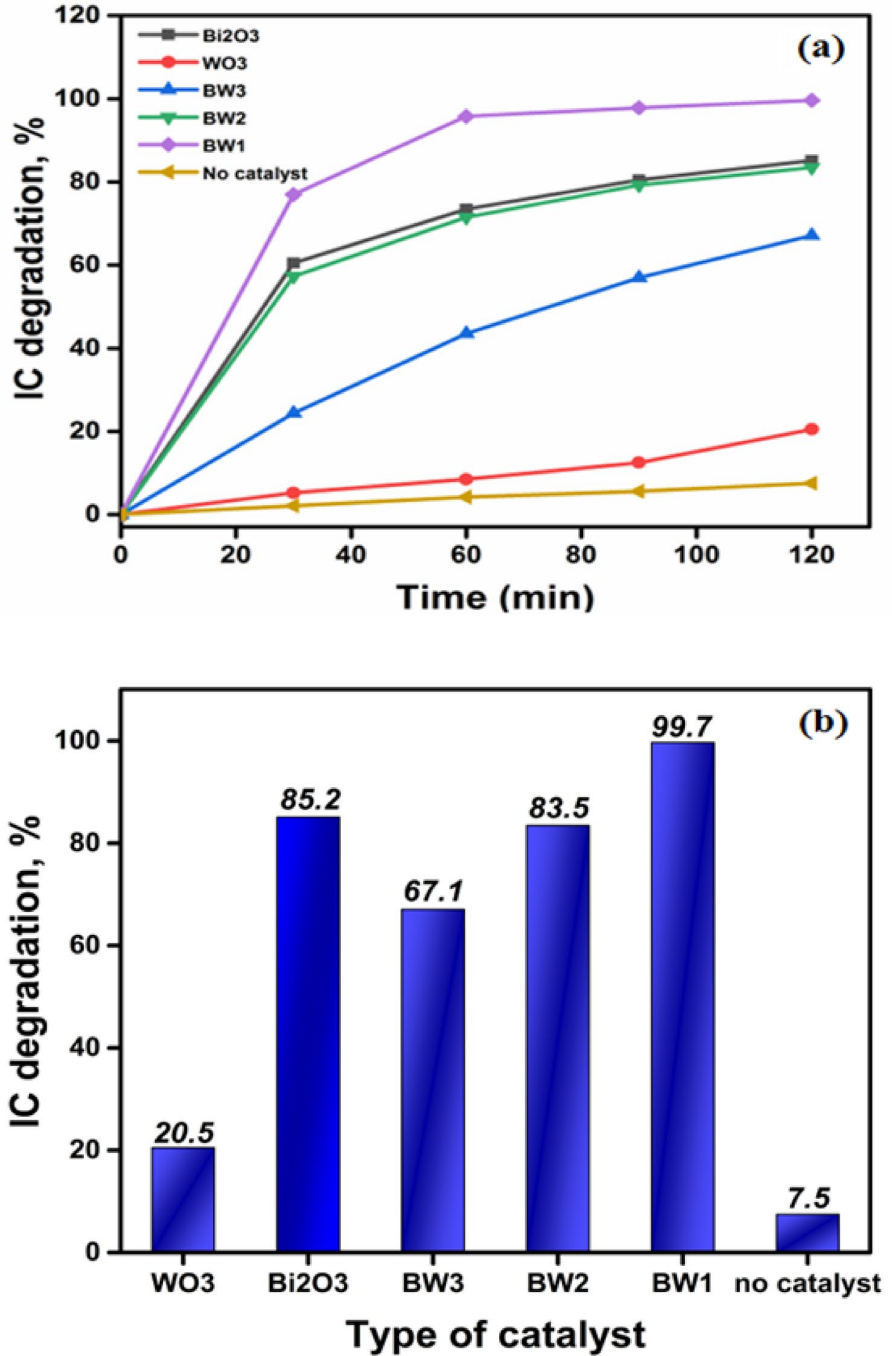
Comparison between the performance of different catalysts in photo-degradation of Indigo carmine (IC) dye (Conc. = 20 ppm, pH = 7.5 and 0.9 g/L catalyst) dyes under UV-A irradiation.

The fabricated materials were analyzed for their photocatalytic-characteristics by observing the degradation of Indigo carmine (IC) dye under UV-A irradiation, as depicted in Fig. 10a,b. The matching UV-visible absorption spectra can be seen in Supplementary Information (Fig. S1). The photoactivities of the samples in degrading IC dye diminish in the following order: BW1 > Bi_2_O_3_ > BW2 > BW3 > WO_3_. To conduct an additional screening test, photolysis was carried out by activating the light source without the presence of any photocatalysts. The elimination efficiency after 2 h was approximately 8%. Regarding the effect of pollutant adsorption on photocatalysis, we would like to point out that the adsorption capacity of the applied catalysts is relatively small, with a maximum adsorption percentage of no more than 7%. As a result, the impact of pollutant adsorption on the overall photocatalytic degradation process is minimal. However, the degradation percentage was evaluated after irradiation, i.e., during the photoreaction step, following the dark adsorption phase. The pure WO_3_ NPs exhibited the lowest degrading efficiency, ~ 21%, after 2 h. However, efficiency is significantly enhanced by the incorporation of Bi_2_O_3_ and the creation of composite heterostructures. The BW1 composite demonstrated the highest photodegradation performance level in the IC dye, achieving an efficiency of 99.7%. Generally, the impregnation of Bi_2_O_3_ to WO_3_ in the synthesized composites, 1:1 in BW2, 0.5:1 in BW3, and 1.5:1 in BW1 improved the photocatalytic activity of the resulting composite in degrading IC dye. Including an excessive amount of Bi_2_O_3_ in the composition of the (BW1; Bi_2_O_3_/Bi_2_WO_6_) catalyst reduces the recombination rate of electron-hole pairs. Consequently, this increases the involvement of many electron-hole pairs in the photocatalytic-reaction, resulting in enhanced catalytic activity. This observation aligns with the findings documented in various investigations in the literature^10,106^. As a result, the BW1 composite was chosen for additional studies to optimize photocatalytic-activity features.

#### Effect of catalyst dose

**Fig. 11:**
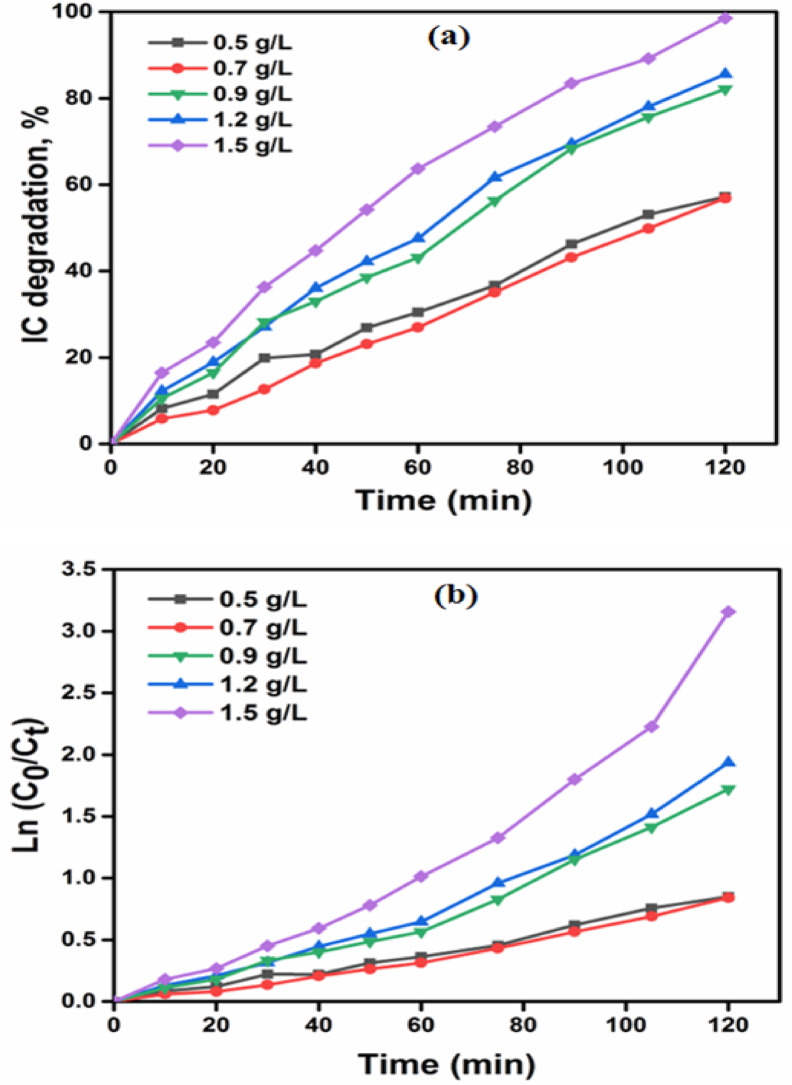
Degradation (%) of IC dye (50 ppm) catalyzed by different doses of BW1 composite under UVA-irradiation (**a**) and plots of ln(C_0_/C_t_) versus time for IC dye degradation (**b**).

The impact of various doses of BW1 composite (ranging from 0.5 to 1.5 g L^− 1^) on its efficacy in degrading IC dye is illustrated in Fig. 11, along with their respective UV-visible absorption spectra in Fig. S2. The current degradation process occurs under UV-A light irradiation at varied time intervals for an initial concentration of 50 ppm of IC dye. In general, the degradation percentage of IC dye increased as the amount of photocatalyst increased at different intervals of reaction, as shown in Fig. 11a. The degradation percentage achieved a maximum of 98.9% after 120 min when an increased quantity of catalyst, specifically 1.5 g/L, was used. In addition, a pseudo-first-order model was employed to establish a relationship between the data on photo-degradation reactions of IC dye and the various catalyst doses of BW1 composite. The rate constant k (min^− 1^) was determined by calculating the slope of the natural logarithm of the initial concentration divided by the concentration at a given time (i.e., ln C_0_/C_t_), plotted against time (t) as presented in Fig. 11b. The degradation of IC dye over BW1 composite using different doses was well described by the pseudo-first-order model, with an R^2^ value of approximately 0.97. The maximum degradation rate constant (kapp) for IC dye was determined to be 0.021 min^− 1^, using a concentration of 1.5 g/L of BW1 composite (Table 3).

**Table 3:** The apparent rate constants (k, min^− 1^) and regression coefficients (R^2^) obtained from the pseudo-first-order kinetic model for the photocatalytic-degradation of IC dye, using various dosages of the BW1 catalyst and different light sources.

Catalyst dose (g/L)	K_app_ (min^− 1^)	*R* ^2^
0.5	0.005	0.991
0.7	0.006	0.990
0.9	0.011	0.988
1.2	0.015	0.985
1.5	0.021	0.973
Light source		
UV-A	0.011	0.967
UV-B	0.002	0.997
Hg-lamp	0.007	0.978
LED-lamp	0.001	0.955

#### Effect of light source

**Fig. 12:**
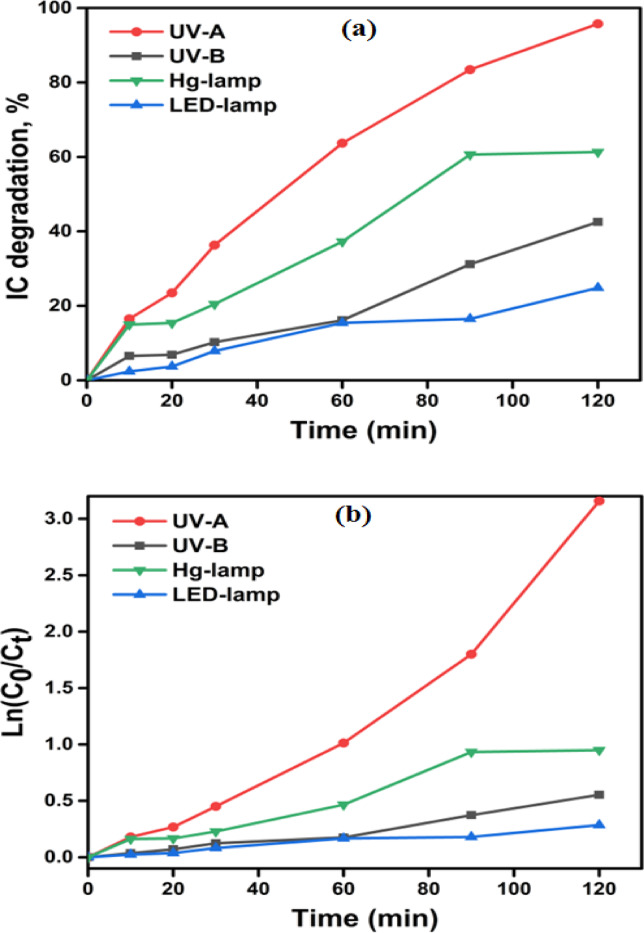
Degradation (%) of IC dye (50 ppm) catalyzed by BW1 composite (1.5 g/L) under different light sources (**a**) and plots of ln(C_0_/C_t_) versus time for IC dye degradation (**b**).

The impact of different light sources on the photocatalytic–degradation of IC dye using the BW1 catalyst was demonstrated in Fig. 12a, and its related UV-visible absorption spectra are shown in Fig. S3. Four distinct light sources were utilized: UV-A (λ = 365 nm), UV-B (λ = 256 nm), LED-lamp, and Hg-lamp. The degradation efficiency rate is ordered as: UV-A > Hg-lamp > UV-B ≈ LED in degrading IC dye within 2 h of irradiation. Generally, the photocatalytic degradation of IC dye is greater when exposed to UV irradiation compared to visible irradiation at various time intervals. This phenomenon can be explained by the fact that UV-A light possesses greater energy than visible-light, allowing UV light to readily permeate the catalyst reaction slurry, which leads to greater activation of the catalyst, creating a higher number of electrons and holes^107^. Despite the high energy of UV-B radiation, the degradation efficiency is relatively low. This observation demonstrates that increasing the photon flux is insufficient if it does not align with the effective band gap of the photocatalyst^108^. However, as depicted in Fig. 12b, the pseudo-first-order model accurately represented the degradation of the IC dye by BW1 under the applied light sources, indicating that the degradation process over the BW1 catalyst under both UV and visible irradiation occurs through an identical chemical pathway. The degradation rate constants were determined as follows: 0.011 min^− 1^ for UV-A, 0.007 min^− 1^ for Hg-lamp, 0.002 min^− 1^ for UV-B, and 0.002 min^− 1^ for LED-lamp (Table 3).

#### Effect of initial pH

**Fig. 13:**
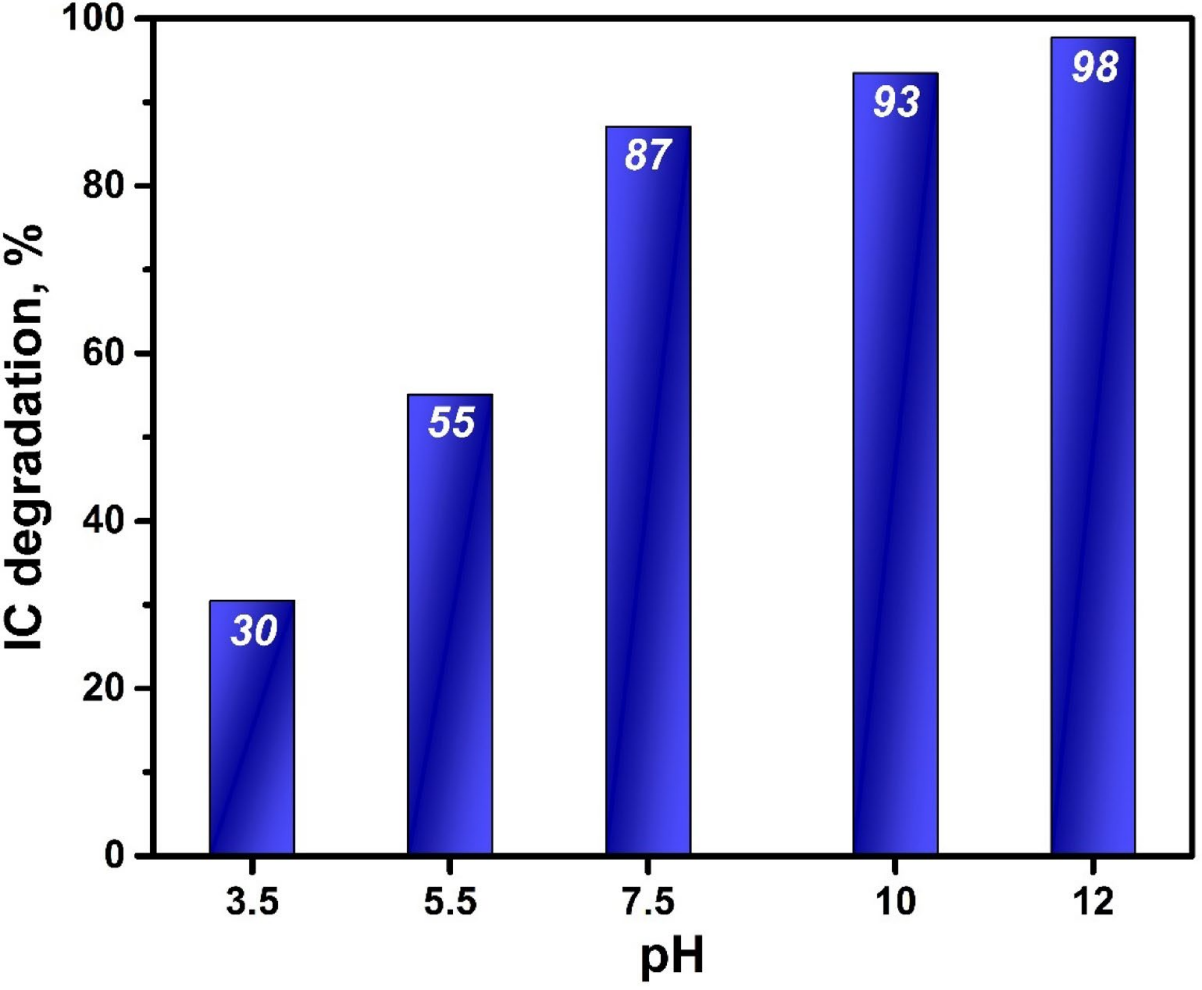
The effect of pH on photodegradation of IC dye (50 ppm) over BW1 composite (0.9 g/L catalyst, 2 h) under UV-A irradiation.

**Fig. 14:**
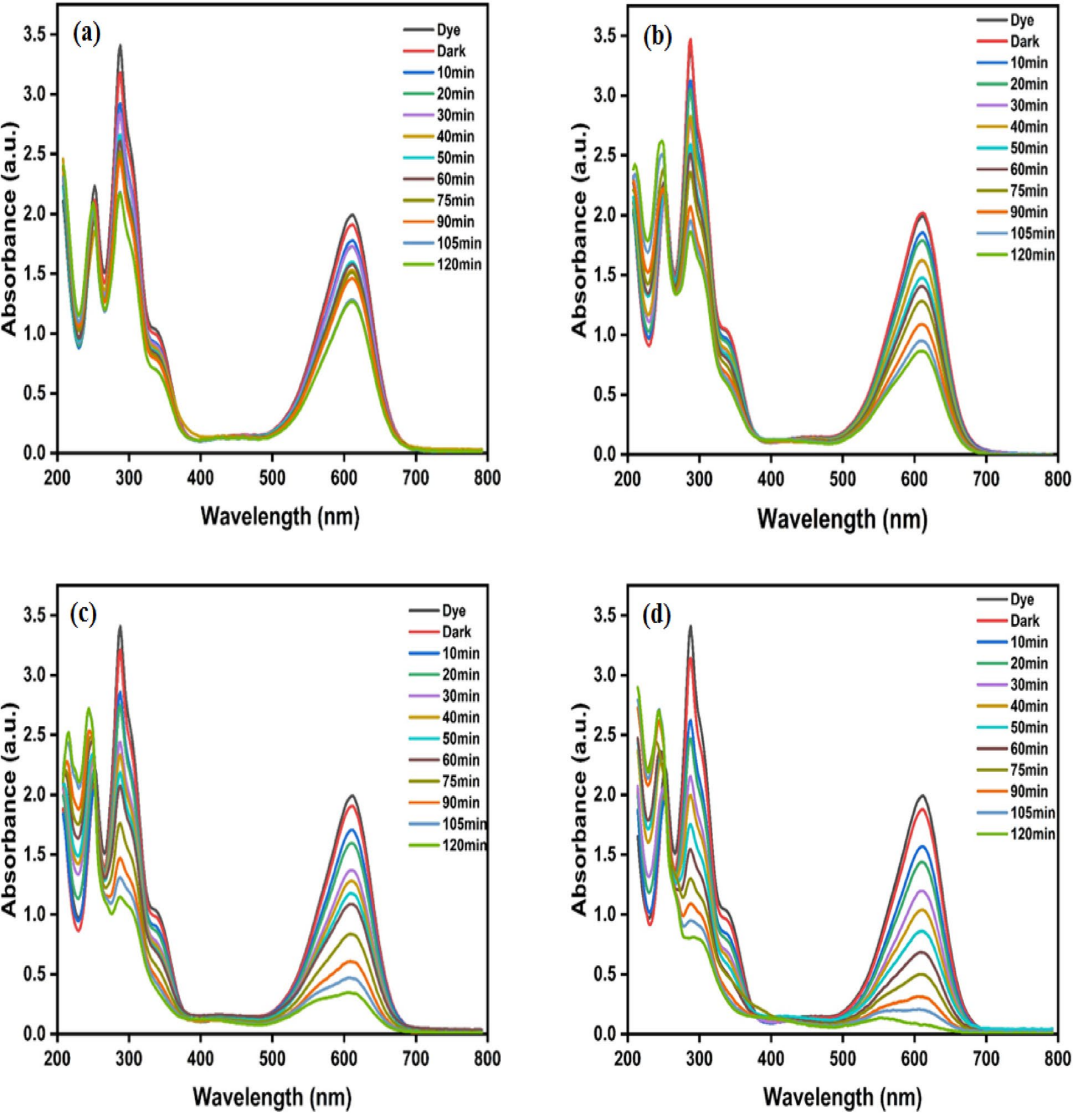
UV-visible absorption spectra of IC dye (50 ppm) catalyzed by BW1 catalyst (1.0 g/L) at different pH values; (**a**) 3.5, (**b**) 5.5, (**c**) 7.5, and (**d**) 10 under UV-A irradiation.

The impact of pH on the photocatalytic-degradation of IC dye using the BW1 composite was illustrated in Fig. 13 and its related UV-visible absorption spectra in Fig. 14. The data shows that when the pH of the solution increased, the percentage of photodegradation of IC dye over BW1 also increased. The highest degradation level was achieved at a pH of 12 after 2 h. Given that IC is an anionic dye, it was anticipated that the degradation would decrease as the pH of the dye solution increased, and the dye removal would be generally lower in alkaline pH solutions. However, the XPS results can provide insight into the potential cause of the existence of oxygen vacancies within the lattice of the BW1 composite. In an alkaline dye solution, the presence of hydroxide ions can induce ionization of these groups, forming negatively charged oxygen groups^109^. Therefore, we can hypothesize that the photoinduced positive hole (h^+^) on the surface of BW1 is likely to react with these ionized negative oxygen groups. This also results in a restriction of the diffusion of holes towards the interface between the composite and the reaction solution, impeding the recombination of electrons (e^−^) and holes (h^+^)^110^. Furthermore, the higher concentration of OH^−^ ions, as opposed to H^+^ ions, in the reaction medium can enhance the creation of more reactive oxygen species (ROS) that facilitate the photo-degradation process^111^. Consequently, the rate of indigo degradation over BW1 showed a consistent increase as the pH increased. This behavior is similar to observations documented in prior studies^10,112^. However, the natural pH value (7.5–8) was selected for all experiments and further studies due to its ease of application in treating aqueous media without prior treatments.

#### Trapping experiments and photocatalytic mechanism

**Fig. 15:**
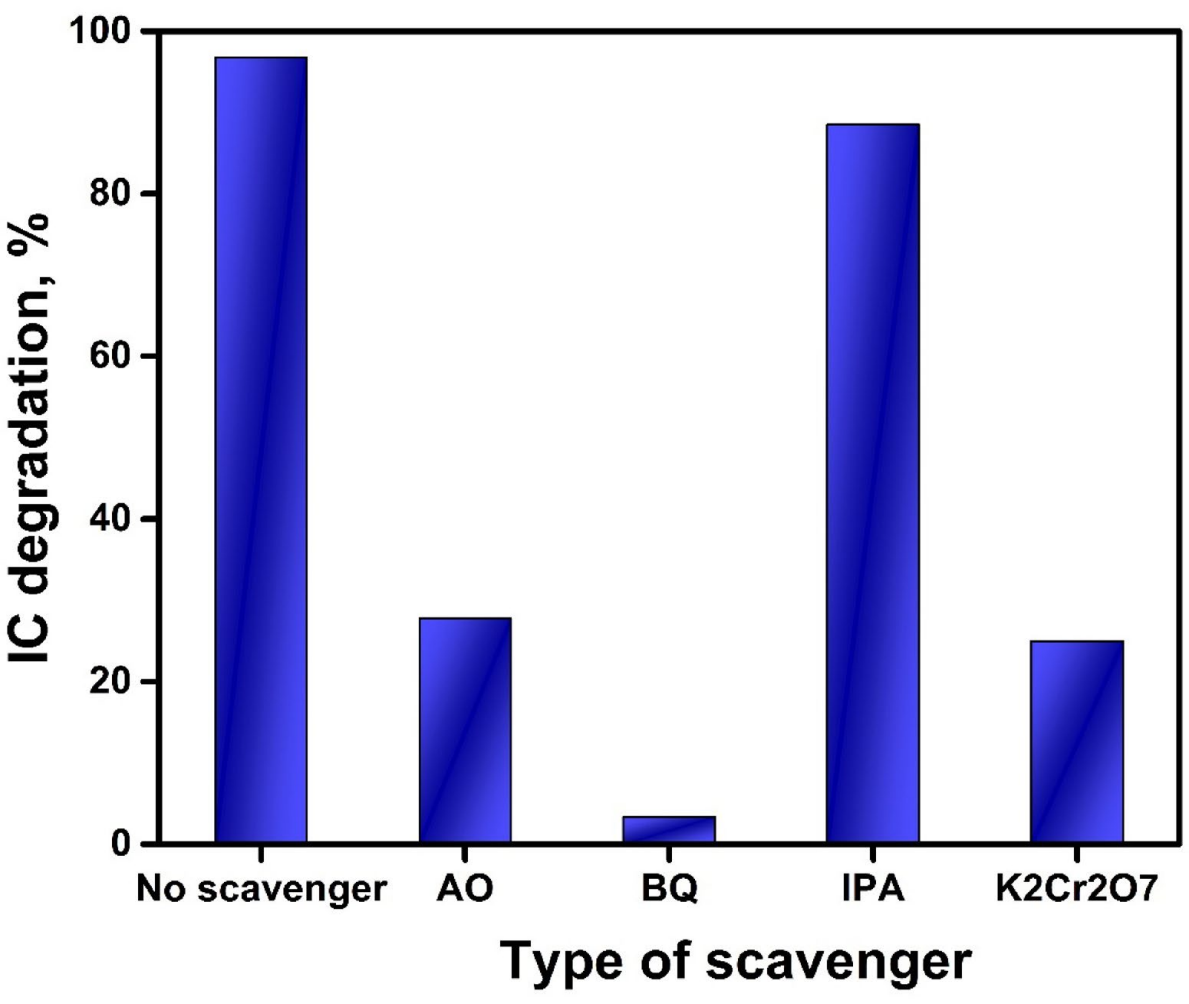
The effect of scavengers on photodegradation of IC dye over BW1 composite.

Experiments to confine active species for the degradation process were carried out to clarify the photocatalytic mechanism of the most potent BW1 catalyst. The scavengers like Benzoquinone (BQ), ammonium oxalate (AO), potassium dichromate (K_2_Cr_2_O_7_), and isopropyl alcohol (IPA) were employed to effectively suppress the activity of superoxide radicals (·O^2−^), photo-holes (h^+^), photoelectrons (e^−^), and hydroxyl radicals (·OH), respectively^113^. The results are portrayed in Fig. 15 and Fig. S4. The results showed that the photodegradation percentage of IC dye on BW1 decreased from 96.7 to 88.5%, 3.3%, 24.9%, and 27.7% with the inclusion of IPA, BQ, K_2_Cr_2_O_7_, and AO, respectively. Thus, it can be deduced that the superoxide radicals play a key role in the degradation process, while the photo-induced electron-hole pairs have a subordinate impact and hydroxyl radicals have a minimal effect. This also suggests that photogenerated holes are not solely the cause of the photodegradation of IC dye.

**Fig. 16:**
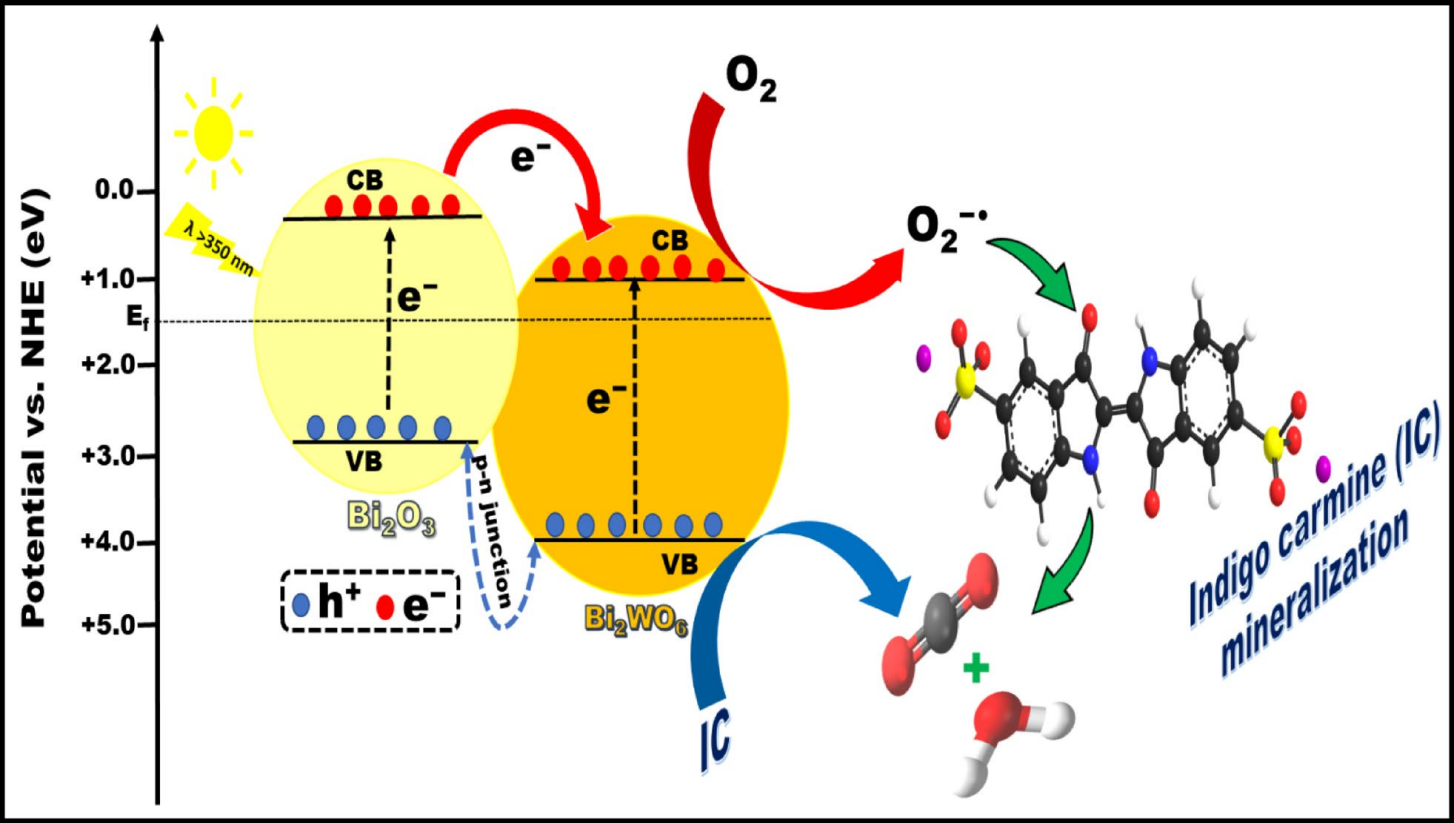
Schematic diagram of charge separation and degradation mechanism of IC dye on the irradiated BW1 composite.

Generally, the type-II heterojunction and Z-scheme structure are the two factors that determine how charges are separated between Bi_2_O_3_ and Bi_2_WO_6_^103^. Upon exposure to visible-light, Bi_2_O_3_ with a narrow bandgap becomes activated, generating photoelectrons and holes. The electrons in the conduction-band of the p-type Bi_2_O_3_ are transferred to the conduction band of the n-type Bi_2_WO_6_ since the Fermi energy level (E_f_) is closer to the CB of Bi_2_WO_6_, while the holes stay fixed in the valence-band of Bi_2_O_3_^38^. This is advantageous for minimizing their recombination. The electrons produced by light were captured by O_2_, forming O_2_^−•^ and H_2_O_2_, then generating hydroxyl radicals^114^. Besides, the photogenerated holes in Bi_2_O_3_ strongly oxidize unsaturated organic contaminants, causing them to be mineralized into CO_2_ and H_2_O^115^. The role of the photogenerated holes in the degradation of organic compounds over the Bi_2_O_3_/Bi_2_WO_6_ catalyst has been well established. Also, the migration rates of the photogenerated electrons and holes are accelerated by the internal electric field within the Bi_2_O_3_/Bi_2_WO_6_ heterojunctions, significantly enhancing the photocatalytic activity. After carefully analyzing the experimental results, we have formulated the most likely type-II heterojunction degradation mechanism of IC dye under visible-light irradiation, as represented in Fig. 16. This proposed mechanism is very consistent with many previous studies documented in literature^98,103,116^.

#### Mineralization of dye

The chemical oxygen demand (COD) is a way to indirectly determine the total organic compounds present in wastewater by measuring the oxygen required for oxidizing organic matter using a chemical oxidant like potassium dichromate^117^. The ultimate objective of the photocatalytic-degradation of organic of the organic dye molecule to CO_2_ and H_2_O^118^. Through examination of the spectral curve of IC dye through photo-degradation experiments, a gradual decline in the dye’s absorption over time was observed across the entire absorption spectrum, without any shifting or new peaks emerging or a significant increase in absorption at any specific wavelength. The reduction in absorbance of the IC dye solution over time is primarily caused by photodegradation and dye conversion into CO_2_ and H_2_O, as documented in various previous research studies^94,119–121^. Nonetheless, assessing mineralization solely through spectrophotometric methods is not feasible. Hence, COD was utilized in current research. Table 4 provides the (CODi) and (CODf) values for IC dye during photocatalytic degradation at various time intervals. The mineralization efficiency of Indigo carmine (IC) dye reached a maximum of 88.9% after 180 min of treatment with the BW1 catalyst. However, when the initial COD was adjusted to account for dye adsorbed, the effective mineralization efficiency was slightly lower at 88.1%. Thus, the data of COD and UV-Vis are consistent with the confirmation that it is not merely discoloration but that the dye’s mineralization occurs into smaller fragments during photocatalytic-reaction^117^.

**Table 4:** Mineralization efficiencies of IC dye (50 mg/L) over BW1 composite.

Time (min)	COD (ppm)	Mineralization efficiency (M, %)*
Control	495	0.0
Adsorption (dark)	463	6.5
30	369	25.4 (20.3)
60	329	33.5 (28.9)
90	286	42.2 (38.2)
120	179	63.8 (61.3)
150	105	78.8 (77.3)
180	55	88.9 (88.1)

*Mineralization efficiency values in parenthesis corrected to COD % adsorption.

#### Reusability of catalyst

**Fig. 17:**
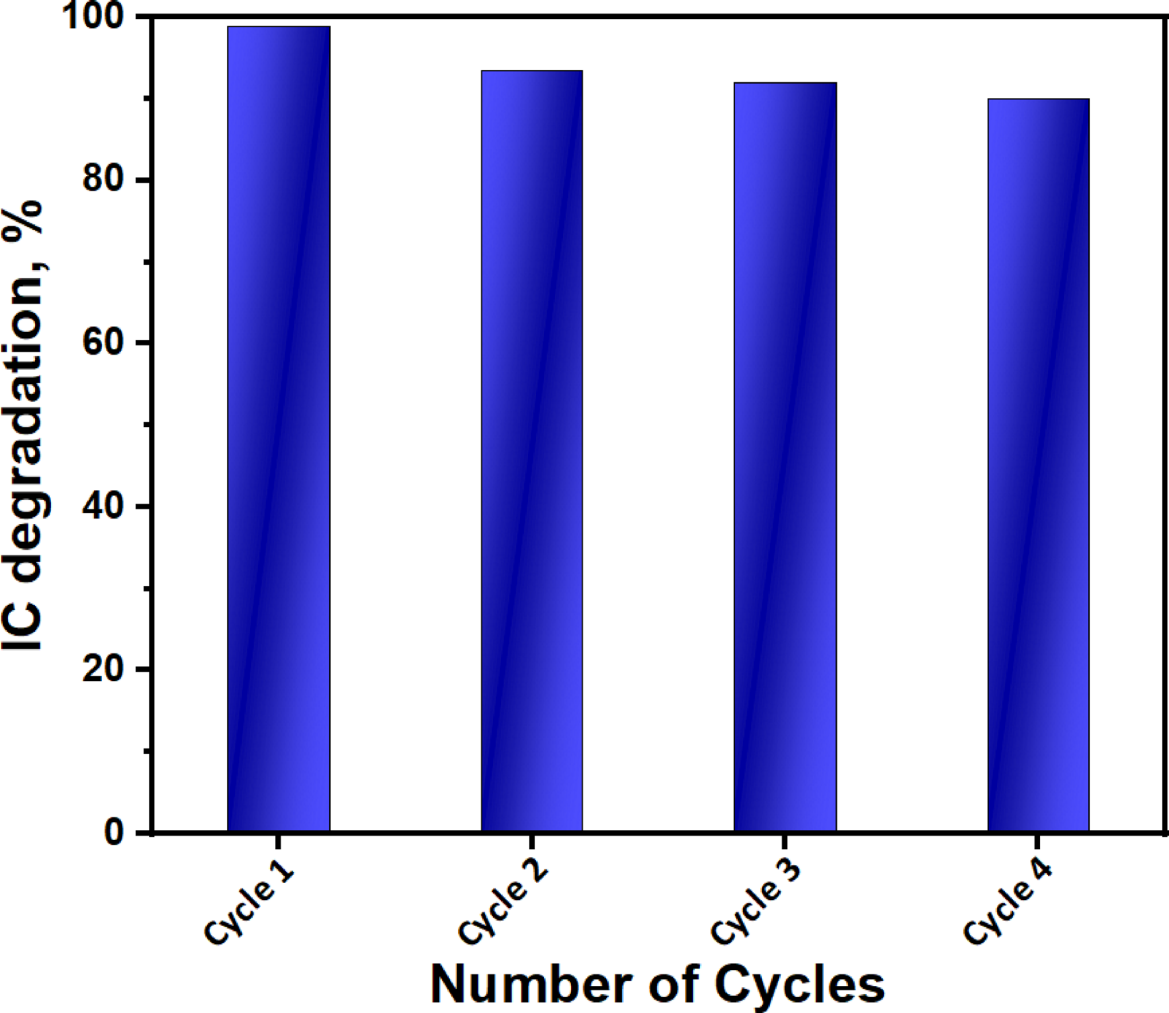
The recycled degradation (%) of IC dye over BW1 composite under UV-A irradiation.

**Fig. 18:**
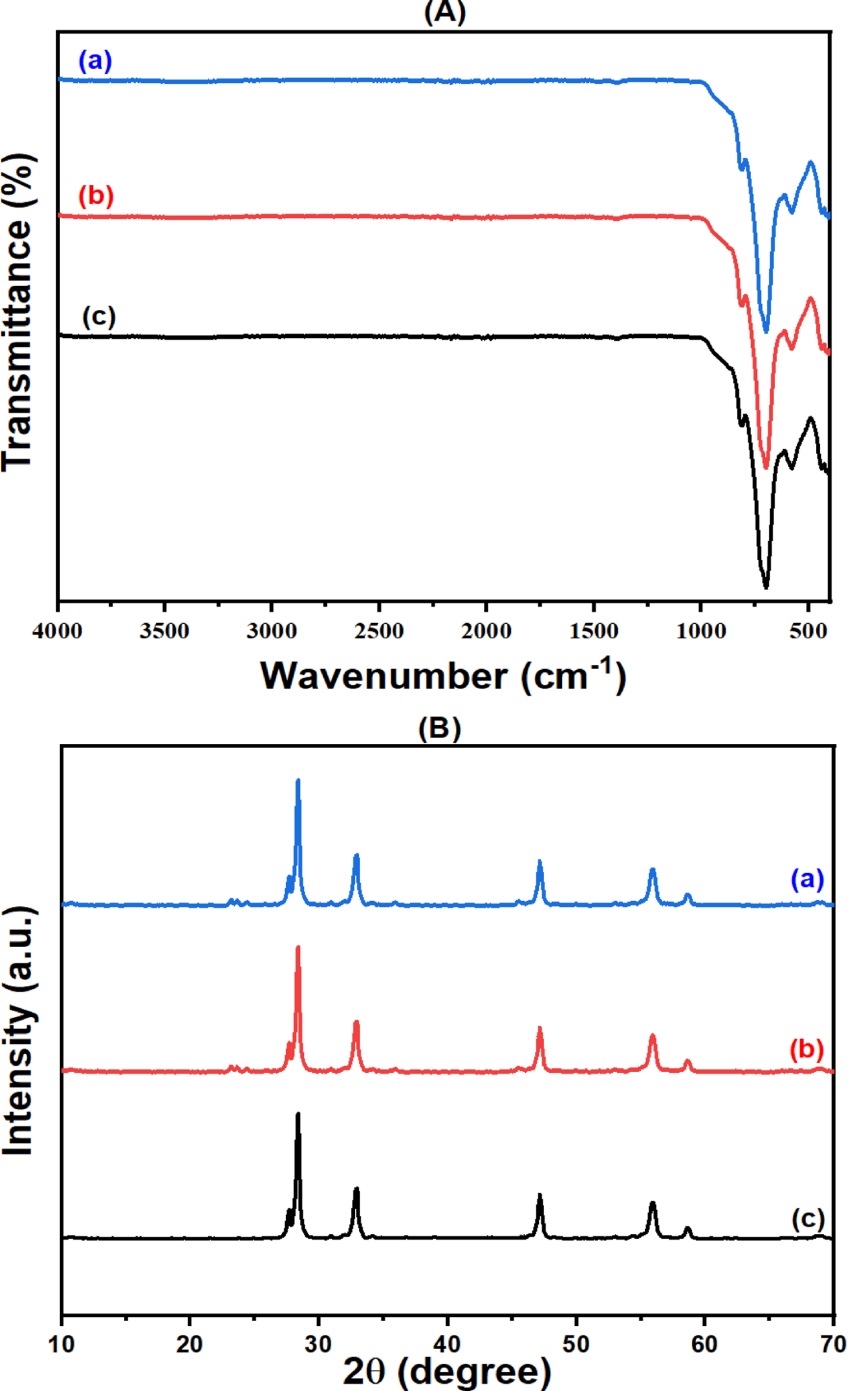
Repetitive FTIR spectra (**A**) and XRD patterns (**B**) of the recycled BW1 composite operated on IC dye: (a) before degradation, (b) after 1st cycle, and (c) after 4th cycle.

This study examined the photocatalytic-stability of the optimized BW1-catalyst in the degradation of IC dye after four consecutive cycles (Fig. 17). The tests were conducted with an initial dye concentration of 50 ppm, a catalyst dosage of 1.5 g/L, and an irradiation period of 120 min at room temperature under UV-A irradiation. As shown in Fig. 18, the BW1 photocatalytic reactivity indicates that approximately 90% of the dye is mineralized throughout the four cycles, indicating significant reusability. The repetitive FTIR-spectra (Fig. 18A) and PXRD-pattern (Fig. 18B) of the recycled nanocomposite were analyzed. The repetitive spectra of the recycled composite (BW1) demonstrate the presence of the primary characteristic peaks for the synthesized composite across four successive cycles, showing its structural stability.

#### Comparison with other catalysts

Importantly, based on our literature review, no prior studies have reported the use of Bi_2_O_3_/Bi_2_WO_6_ nanocomposites specifically for the degradation of Indigo Carmine dye under visible light irradiation. This underlines the novelty of our work and demonstrates the potential of our composite as an efficient visible-light-driven photocatalyst for this organic pollutant. The Bi_2_O_2_/Bi_2_WO_6_ composite exhibited near-complete degradation (~ 100%) of Indigo Carmine (IC) dye after 120 min of visible light exposure. This high efficiency ranks among the best in our dataset (Table S1) and is on par with other catalysts used for similar dye degradation.

## Conclusions

This study explores the role of Bi^3+^ impregnation in enhancing the visible-light response of WO_3_ nanoparticles and discusses its implications for the design of efficient inorganic photocatalysts. Highly efficient Bi_2_O_3_/Bi_2_WO_6_ nanocomposites were synthesized through a facile in-situ hydrothermal method, employing WO_3_ as a substrate and followed by annealing. This approach created a tightly bonded interface between the two semiconductors, facilitating efficient charge carrier transfer and separation. The resulting Bi_2_O_3_/Bi_2_WO_6_ composite demonstrated significantly enhanced photocatalytic-activity for hazardous indigo carmine (IC) dye degradation under visible-light irradiation, with the optimal performance observed for the 1.5 wt% Bi_2_O_3_:Bi_2_WO_6_ catalyst. The superior photocatalytic activity resulted from the synergistic effect between Bi_2_O_3_ and Bi_2_WO_6_, which broadened the visible-light absorption range and separated charge efficiently at the heterojunction interface. Radical and hole-trapping experiments revealed that the photoinduced-electrons and superoxide-radicals were the main reactive species that caused the degradation process. This study presents a promising strategy for developing novel heterojunction photocatalysts to remove hazardous organic pollutants efficiently.

## Electronic supplementary material

Below is the link to the electronic supplementary material.

Supplementary Material 1

## Data Availability

The datasets used and/or analyzed during the current study available from the corresponding author on reasonable request.
