# Supplementary material for: Hydrothermal synthesis and structural optimization of Bi2O3/Bi2WO6 nanocomposites for synergistic photodegradation of Indigo Carmine dye
Source: Sci Rep. 2025 May 18;15:17260. doi: 10.1038/s41598-025-01925-z (PMC12086239; doi:10.1038/s41598-025-01925-z)
Supplement: Supplementary file 1 — Supplementary Material 1 [file 41598_2025_1925_MOESM1_ESM.docx]

**Supporting information for:**

**Hydrothermal Synthesis and Structural Optimization of** **Bi_2_O_3_/Bi_2_WO_6_ Nanocomposites for Synergistic Photodegradation of** **Indigo Carmine Dye**

Mostafa. A. Sayed^*1^, S.M.A. El-Gamal^1^, M. Ramadan^1^, Fatma. M. Helmy^1^, Alaa Mohsen^2^

^1^Department of Chemistry, Faculty of Science, Ain Shams University, Cairo, Egypt

^2^Faculty of Engineering, Ain Shams University, Cairo, Egypt

***Corresponding Author:** Mostafa Abdellah Sayed ([mostafa_abdellah@sci.asu.edu.eg](mailto:mostafa_abdellah@sci.asu.edu.eg))

***Characterization and Measurements***

The X-ray diffraction analysis (XRD, model Xpert-2000, Philips) of the synthesized samples is obtained at a scanning rate of 5 degrees/minute and a phase size of 0.05 ° using CuKα-radiation in the 2Ө range from (20° - 80°). The sample size and morphological structure were examined using transmission-electron microscopy (TEM). TEM-images were captured using Jeol (JEM-2100 PLUS) at an accelerating voltage of 200kV. Field-emission scanning-electron microscopy (FESEM: ZEISS, model Sigma 300 VP) was applied to monitor the morphology and microstructure. The functional groups of the fabricated samples were detected via Fourier-transform infrared spectroscopy (FTIR: model-Unicam- Mattson-1000). The optimized heterojunction was examined via X-ray photoelectron spectrum (XPS), which was collected on K-ALPHA (Thermo Fisher Scientific, USA) with monochromatic X-ray Al K-alpha radiation ranged from -10 to 1350 eV as the spot size is 400 μm at pressure ≈ 10-9 mbar. The UV–visible spectra are recorded in the 200-800 nm range on a Shimadzu UV-2700 spectrophotometer. The photoluminescence (PL) spectra at room temperature were recorded using a Shimadzu RF 6000 spectrometer at an excitation wavelength of 300 nm.


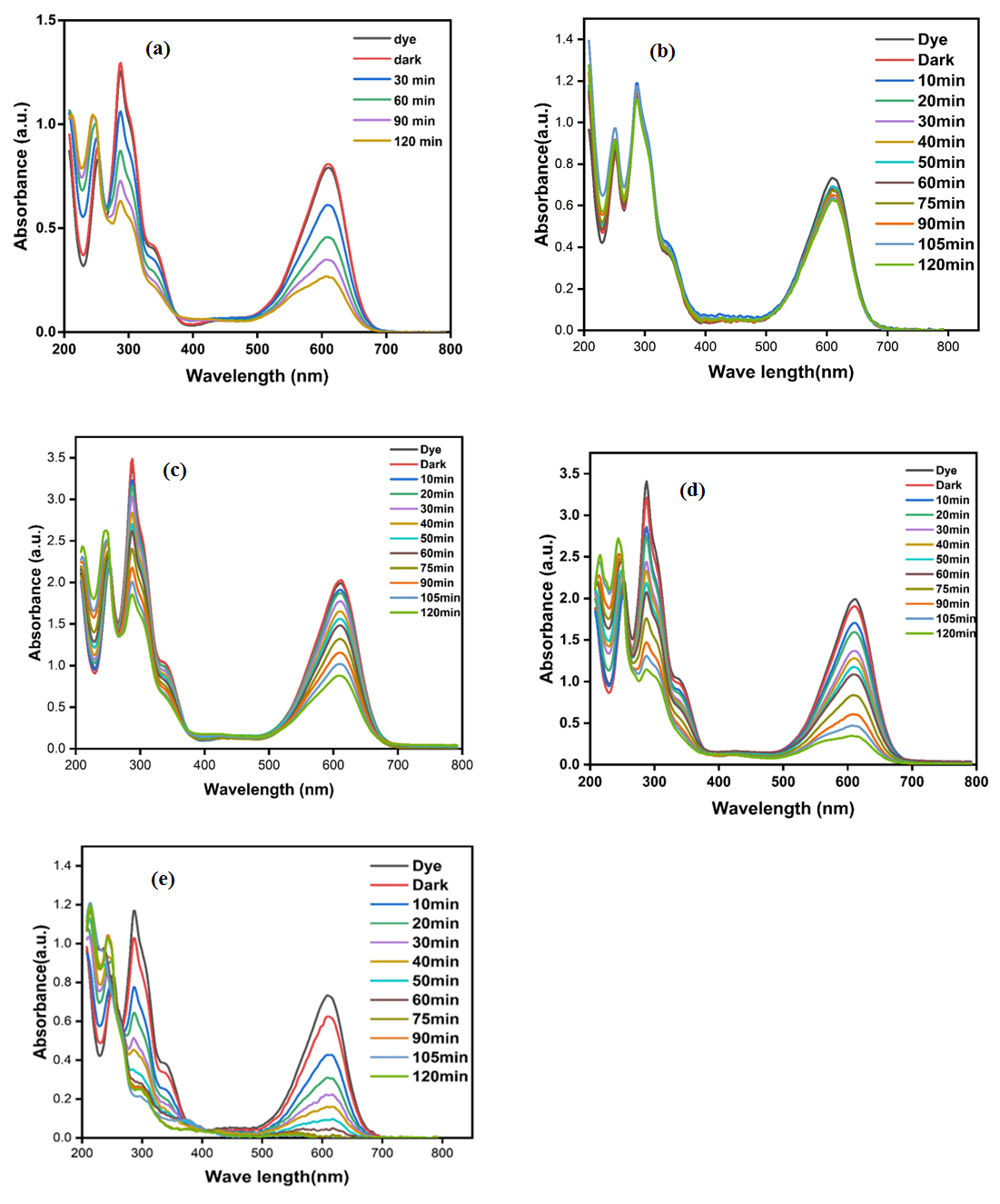


**Fig. S1**. UV-visible absorption spectra of IC dye in the presence of different catalysts: (a) WO_3_, (b) Bi_2_O_3_, (c) BW3, (d) BW2, and (e) BW1. Reaction conditions: 0.5 g/L catalyst under UV-A irradiation (λ=365 nm) at pH=7.5.

***
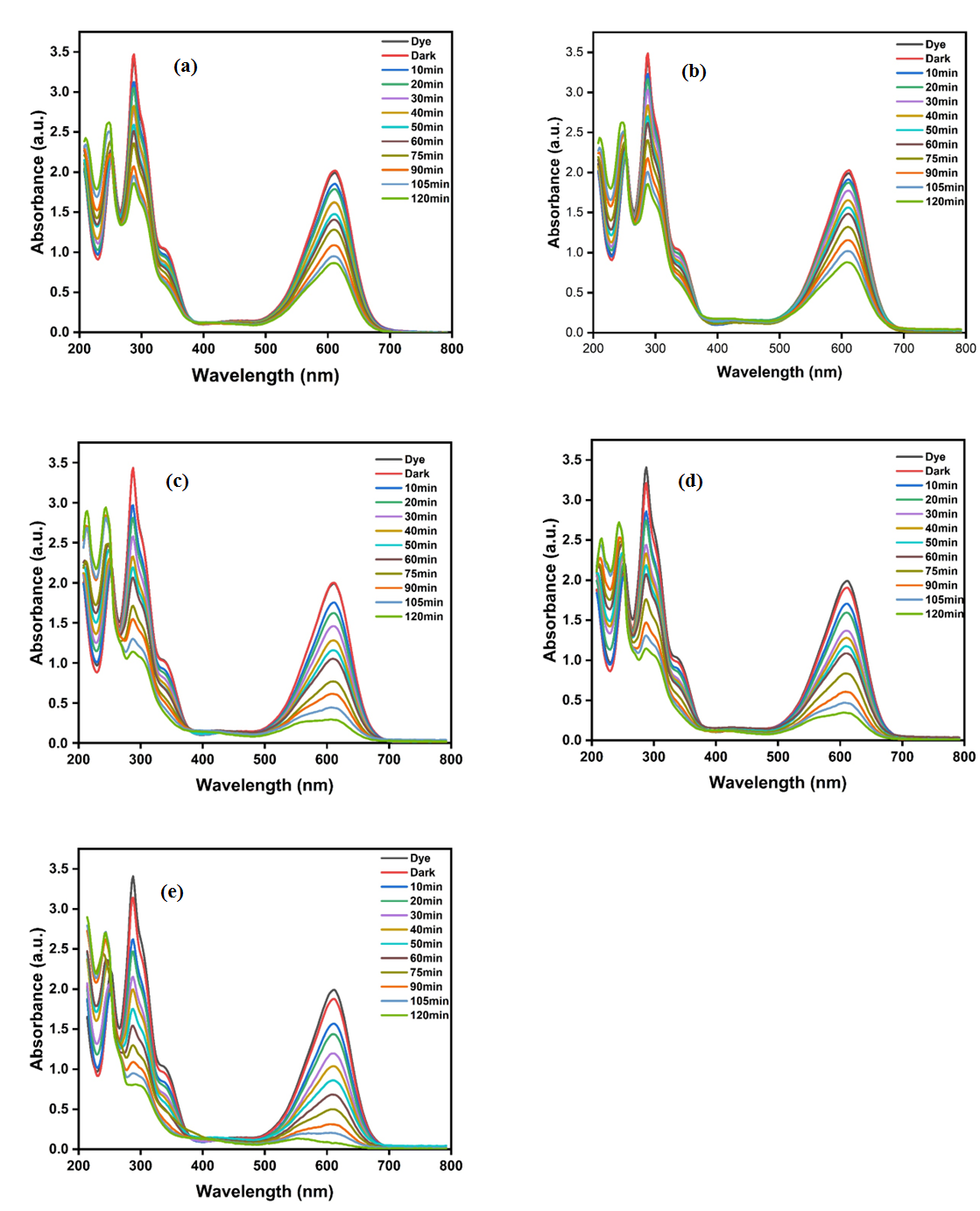
***

**Fig. S2**. UV-visible absorption spectra of IC dye catalyzed by different doses of BW1 catalyst [(a) 0.5 g/L, (b) 0.7 g/L, (c) 0.9 g/L, (d) 1.2 g/L, and (e) 1.5 g/L] under UV-A irradiation (λ=365 nm) at natural pH.

***
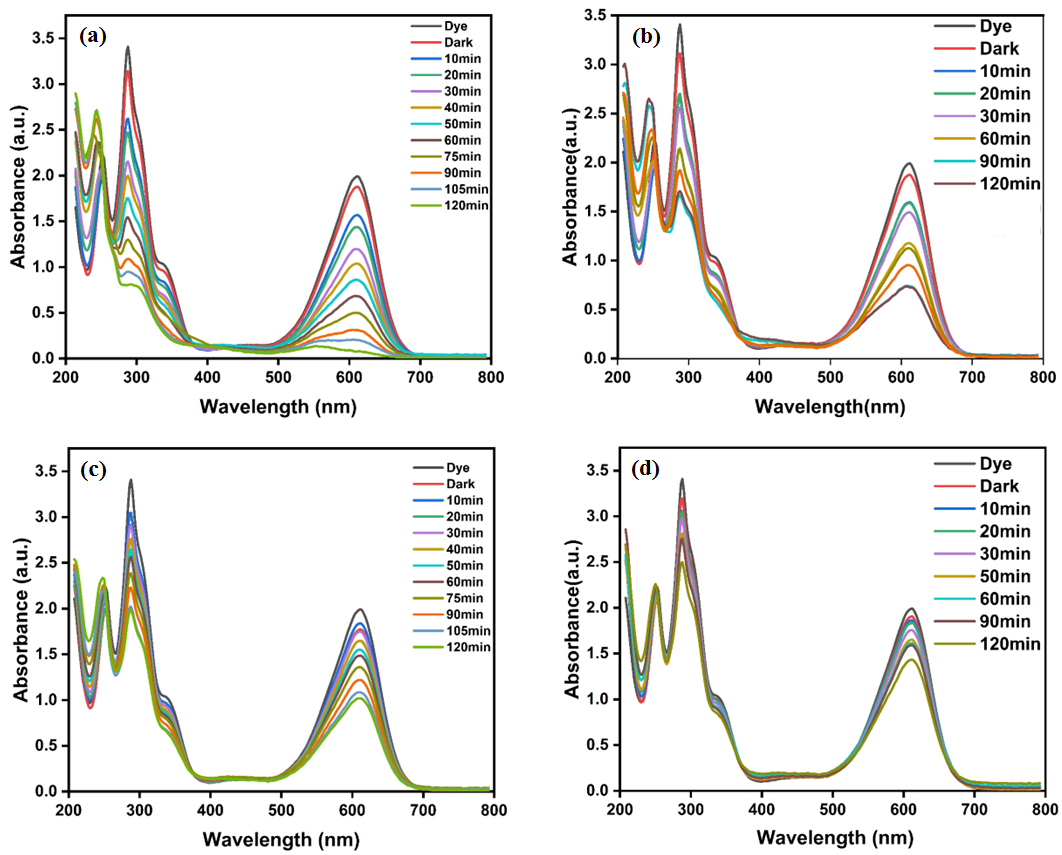
***

**Fig. S3**. UV-visible absorption spectra of IC dye (50 ppm) irradiated by different light sources over BW1 catalyst (1.5 g/L) [(a) UV-A, (b) Hg-lamp, (c) UV-B, and (d) LED-lamp] at natural pH.

***
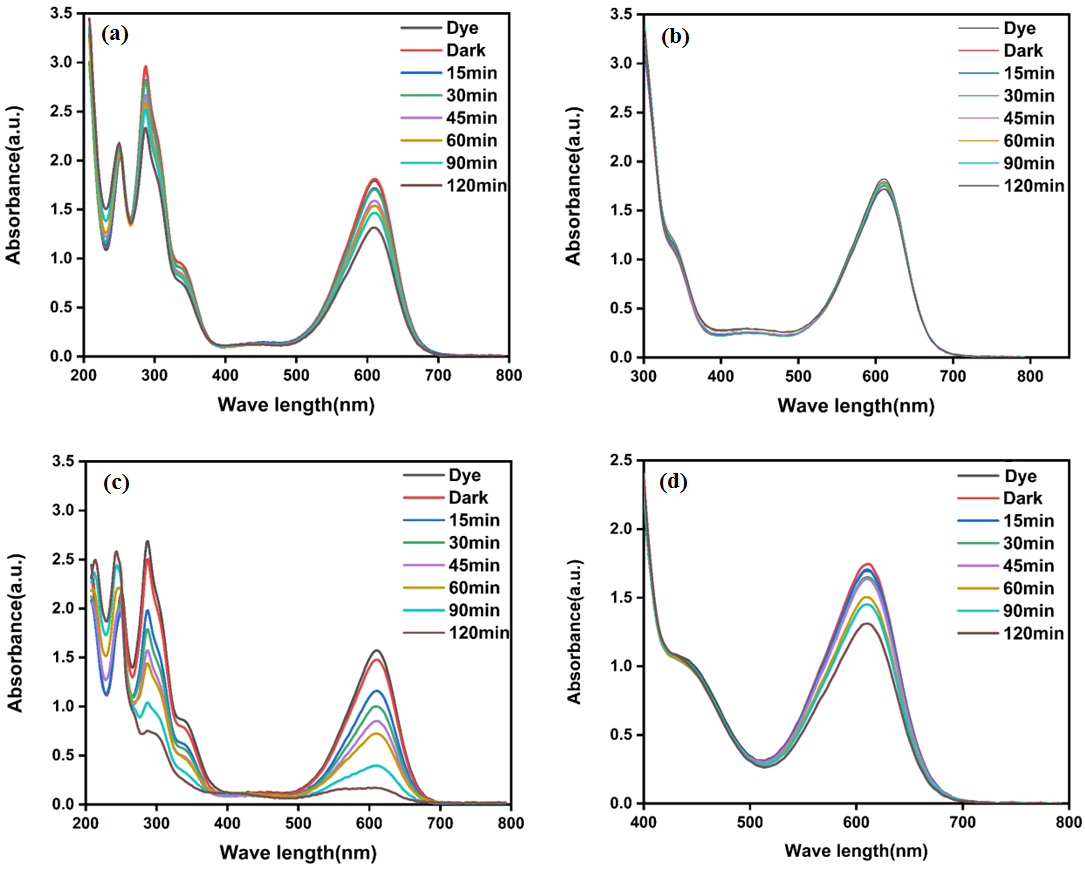
***

**Fig. S4**. UV-visible absorption spectra of IC dye (50 ppm) catalyzed by BW1 catalyst (1.5 g/L) in the presence of different scavengers: (a) ammonium oxalate, (b) benzoquinone, (c) isopropyl alcohol, and (d) potassium dichromate under UV-A irradiation at natural pH.

**Table S1**. Comparison of the efficiency of as-synthesized Bi_2_O_3_/Bi_2_WO_6_ composite with catalysts based on its components reported recently in literature

| **Catalyst** | **Synthesis method** | **Pollutant** | **Degradation (%)** | **Light source** | **Ref.** |
| --- | --- | --- | --- | --- | --- |
| Bi_2_O_3_/Bi_2_WO_6_ | Hydrothermal | Indigo carmine | 99.5 | Visible light | This study |
| Bi₂O₃/WO₃ | Hydrothermal | RhodamineB | 98% | Visible light | [1] |
| Bi₂O₃–Bi₂WO₆ | One-pot hydrothermal | Tetracycline hydrochloride | 85% | Visible light | [2] |
| Bi₂O₃/WO₃ heterojunction | Wet chemical method | Methylene blue | 90% | Visible light | [3] |
| Bi₂WO₆/rGO | Solvothermal | Rhodamine B | 97.6% | Visible light | [4] |
| Bi₂O₃–Bi₂WO₆ nanosheets | Two-step hydrothermal | Bisphenol A | 93% | Visible light | [5] |
| Bi₂O₃/WO₃ heterojunction | Wet chemical method | Methyl orange | 80% | Visible light | [3] |
| Bi₂O₃/WO₃ | Hydrothermal | Crystal violet | 89% | Visible light | [1] |
| Bi₂WO₆–Bi₂O₃ hybrid | Hydrothermal | Tetracycline hydrochloride | 92.5% | Simulated sunlight | [6] |
| Bi₂O₃/WO₃ | Microwave-assisted hydrothermal | RhodamineB | 96% | Visible light | [7] |
| Bi₂O₃-WO₃ heterojunction | Ultrasonic-assisted precipitation | RhodamineB | 91% | Visible light | [3] |
| Bi₂WO₆/Bi₂O₃ composite | Solid-state reaction | Congo red | 87% | Visible light | [8] |
| Bi₂O₃-WO₃ nanocomposite | Hydrothermal | Phenol | 88% | Visible light | [9] |
| BiOBr/Bi_2_WO6 | hydrothermalmethod | RhodamineB | 95% | Visible light | [10] |

**References**

[1] Y.-C. Liang, H.-C. Yang, Boosting photoresposive ability of WO3–Bi2O3 nanocomposite rods via annealing-induced intrinsic precipitation of nanosized Bi particles, Nanotechnology, 32 (2021) 315701.

[2] A. El Aouni, M. El Ouardi, M. Arab, M. Saadi, H. Haspel, Z. Kónya, A. Ben Ali, A. Jada, A. BaQais, H. Ait Ahsaine, Design of Bismuth Tungstate Bi2WO6 Photocatalyst for Enhanced and Environmentally Friendly Organic Pollutant Degradation, Materials, 17 (2024) 1029.

[3] S.P. Adhikari, H. Dean, Z.D. Hood, R. Peng, K.L. More, I. Ivanov, Z. Wu, A. Lachgar, Visible-light-driven Bi 2 O 3/WO 3 composites with enhanced photocatalytic activity, Rsc Advances, 5 (2015) 91094-91102.

[4] Z.M. Alaizeri, H.A. Alhadlaq, S. Aldawood, M.J. Akhtar, M. Ahamed, Bi2O3-Doped WO3 nanoparticles decorated on rGO sheets: simple synthesis, characterization, photocatalytic performance, and selective cytotoxicity toward human cancer cells, ACS omega, 8 (2023) 25020-25033.

[5] M.-S. Gui, W.-D. Zhang, Q.-X. Su, C.-H. Chen, Preparation and visible light photocatalytic activity of Bi2O3/Bi2WO6 heterojunction photocatalysts, Journal of Solid State Chemistry, 184 (2011) 1977-1982.

[6] A. Elaouni, M. El Ouardi, A. BaQais, M. Arab, M. Saadi, H.A. Ahsaine, Bismuth tungstate Bi 2 WO 6: a review on structural, photophysical and photocatalytic properties, RSC advances, 13 (2023) 17476-17494.

[7] S. Kanwal, M. Khan, M. Uzair, M. Fatima, M. Bukhari, Z. Saman, H. Elsaeedy, U. Shahzad, Z.M. Elqahtani, N. Alwadai, A facile green approach to the synthesis of Bi2WO6@ V2O5 heterostructure and their photocatalytic activity evaluation under visible light irradiation for RhB dye removal, Arabian Journal of Chemistry, 16 (2023) 104685.

[8] N.A. Shad, M. Zahoor, K. Bano, S.Z. Bajwa, N. Amin, A. Ihsan, R.A. Soomro, A. Ali, M.I. Arshad, A. Wu, Synthesis of flake-like bismuth tungstate (Bi2WO6) for photocatalytic degradation of coomassie brilliant blue (CBB), Inorganic Chemistry Communications, 86 (2017) 213-217.

[9] B. Yao, G. Zheng, Y. Luan, L. Wang, X. Xing, Y. Wang, Y. Liu, J. He, F. Zhang, Cost-effective Bi2WO6 for efficient degradation of rhodamine B and tetracycline, Journal of Materials Science: Materials in Electronics, 34 (2023) 246.

[10] X. Meng, Z. Zhang, Facile synthesis of BiOBr/Bi2WO6 heterojunction semiconductors with high visible-light-driven photocatalytic activity, Journal of Photochemistry and Photobiology A: Chemistry, 310 (2015) 33-44.
